# Supplementary material for: Structure-guided mutagenesis reveals a hierarchical mechanism of Parkin activation
Source: Nat Commun. 2017 Mar 9;8:14697. doi: 10.1038/ncomms14697 (PMC5347139; doi:10.1038/ncomms14697)
Supplement: Supplementary Information — Supplementary Figures [file ncomms14697-s1.pdf]

Supplementary Figure 1

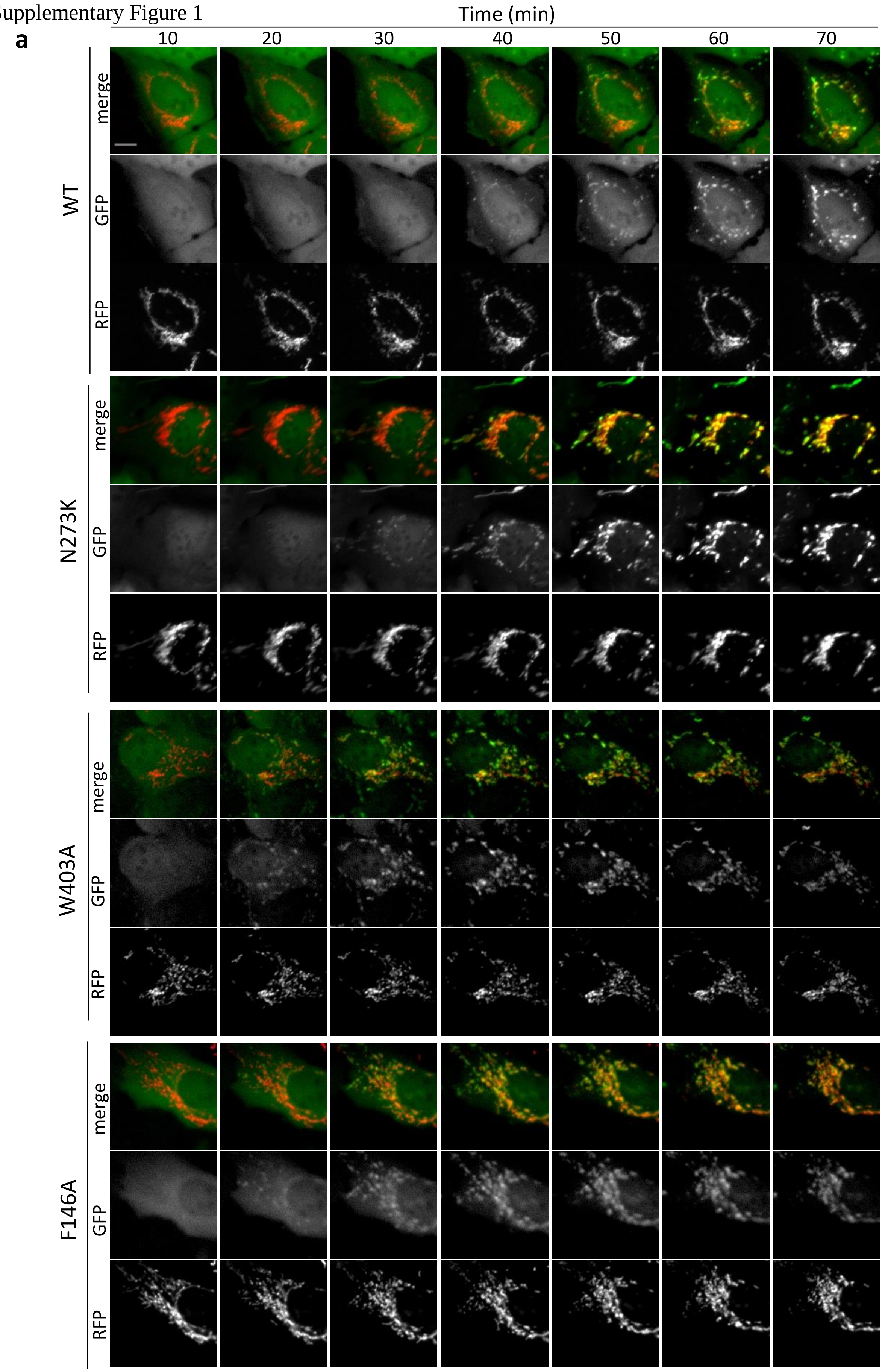

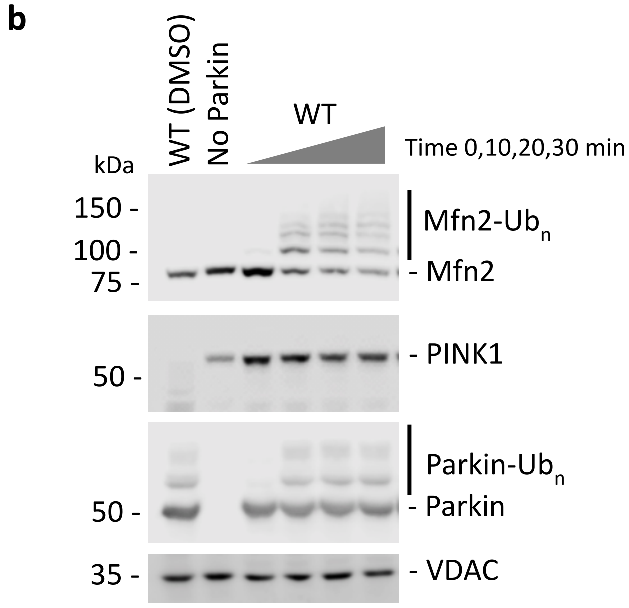

Supplementary Figure 1. Activating Parkin mutations. (a) Time-lapse microscopy showing recruitment of GFP-Parkin mutants to mitochondria upon membrane depolarization with CCCP in U2OS cell stably expressing WT, N273K, W403A, or F146A Parkin were transduced with CellLight© mitochondria-RFP as per manufacture protocol (ThermoFisher Scientific). Images represents 3 replicate experiments with over 300 cells analyzed in each condition. Scale bar: 10 µm. **(b)** Western blot of *in organello* ubiquitination reactions showing that ubiquitination of Mfn2 is dependent on the presence of both PINK1 and Parkin. All *in organello* ubiquitination reactions are performed with CCCP-treated mitochondria unless stated differently.

Supplementary Figure 2

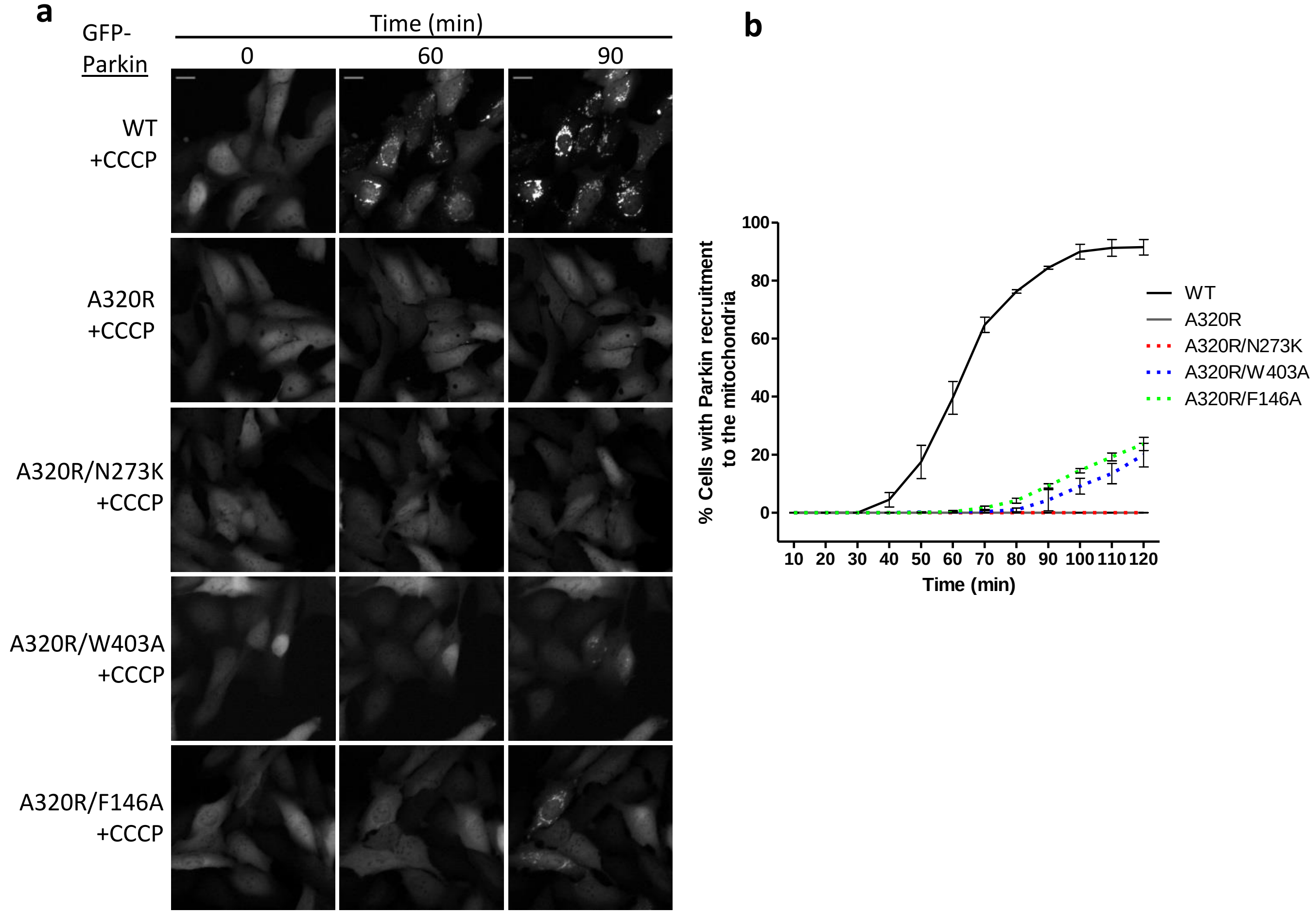

Supplementary Figure 2. Effects of Parkin mutants that disrupt pUb binding on Parkin recruitment. (a) Time-lapse imaging of Parkin recruitment to mitochondria upon treatment with 20 μM CCCP in U2OS cells stably expressing WT, A320R, A320R/N273K, A320R/W302A or A320R/F146A Parkin. Recruitment can be visualized by the appearance of punctate GFP fluorescence. Scale bar: 20 μm. **(b)** Quantification of GFP-Parkin recruitment to the mitochondria. The percentage of cells showing recruitment of GFP-Parkin to mitochondria was determined every 10 mins over a period of 120 mins. The vertical bars represent SEM from two independent experiments.

Supplementary Figure 3

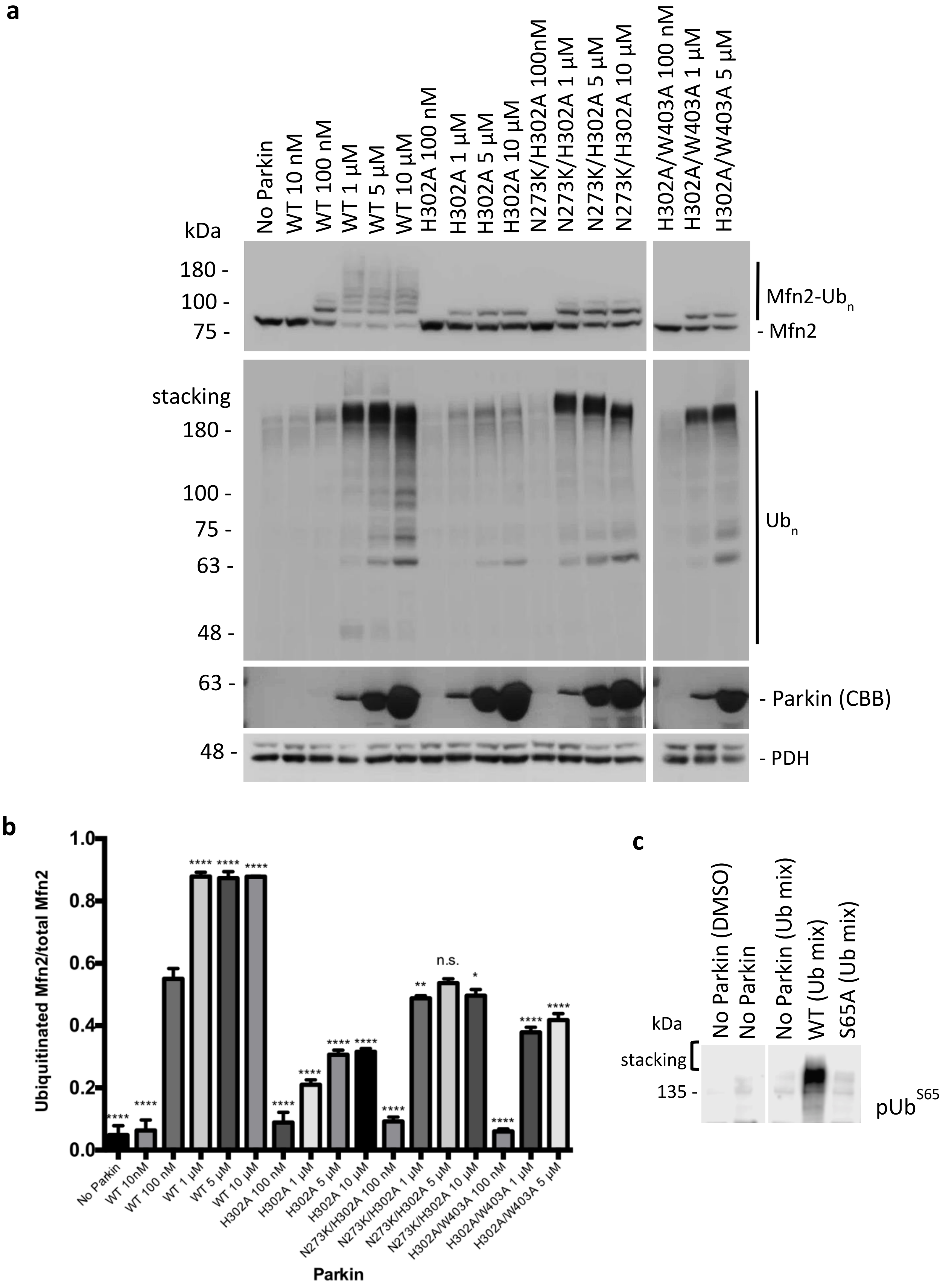

d

e

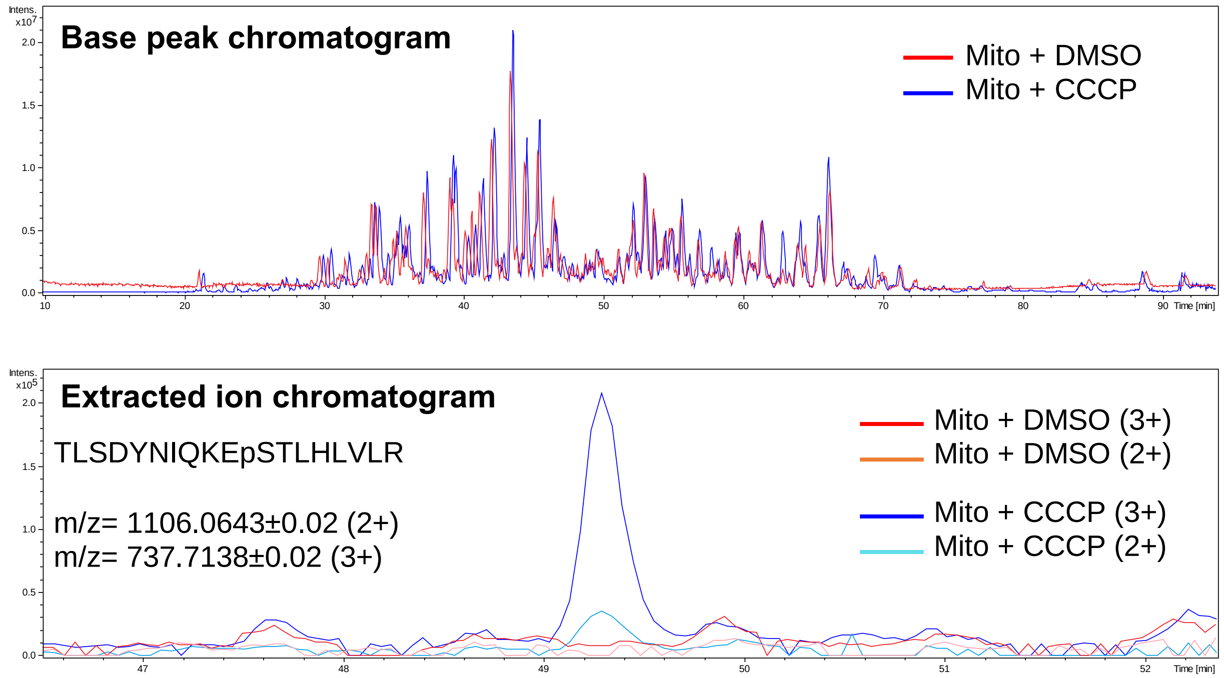

Supplementary Figure 3. Increasing concentration of Parkin mutants defective in pUb binding affect its E3 ligase activity on mitochondria. (a) Western blot of *in organello* ubiquitination reactions using increasing concentrations of Parkin. **(b)** Quantification of Mfn2 ubiquitination signals from three independent experiments. The vertical bars represent SEM. * $P<0.05$; **$P<0.01$; ****$P<0.0001$; n.s., non-significant (one-way ANOVA with Dunnett's test). **(c)** Western blot of *in organello* ubiquitination reactions showing the presence of phosphorylated ubiquitin in samples without Parkin. The Ub mix contains 20nM ubiquitin-activating enzyme 1 (E1), 100nM of ubiquitin-conjugating enzyme 2 (E2), 5µM ubiquitin, 1mM ATP, 5mM MgCl2 and 50µM TCEP, with or without 100nM of recombinant *Rn*Parkin. **(d)** Mass spectrometry base peak chromatograms of isolated polarized (DMSO) and depolarized (CCCP) mitochondria, showing that similar amounts of peptides were loaded on the column. **(e)** Extracted ion chromatograms for the same samples as in (d), showing the doubly and triply-charged ion corresponding to the pUb peptide TLSDYNIQKEpSTLHLVLR. The results show that pUb is detectable in depolarized mitochondria, even in the absence of Parkin.

Supplementary Figure 4

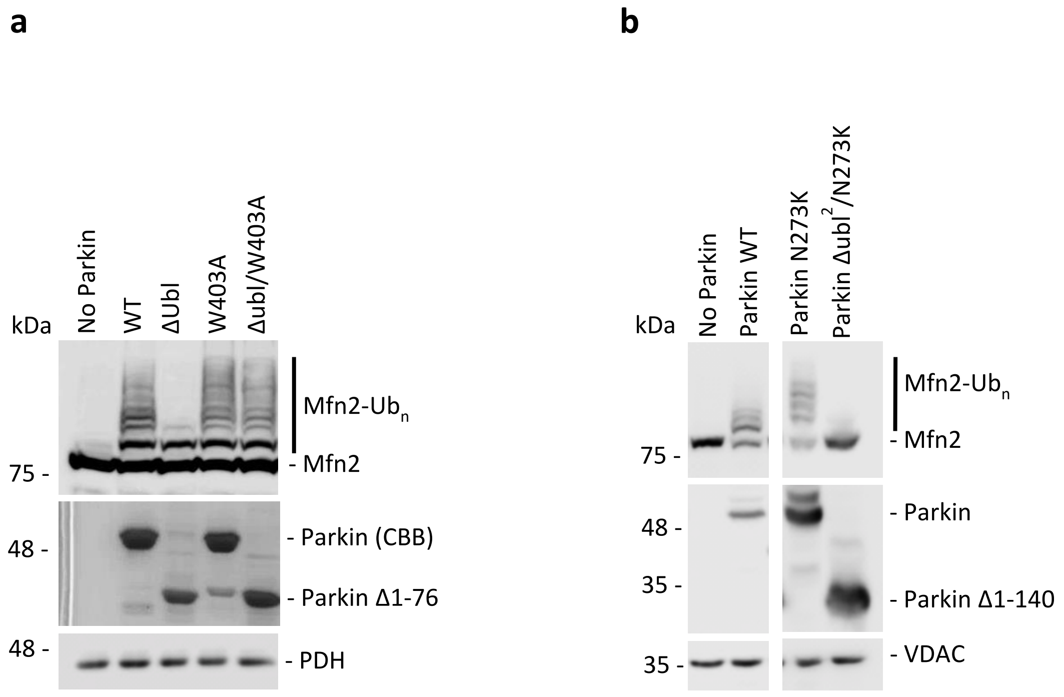

Supplementary Figure 4. *In organello* ubiquitination assay showing that ΔUbl Parkin substrate ubiquitination on mitochondria is independent of the linker between the Ubl and RING0. Western blot of *in organello* ubiquitination assays for the rescue of **(a)** ΔUbl (deletion of residues 1-76) with W403A activating mutation and of **(b)** ΔUbl^2 (deletion of residues 1-140) with N273K activating mutation.

Supplementary Figure 5

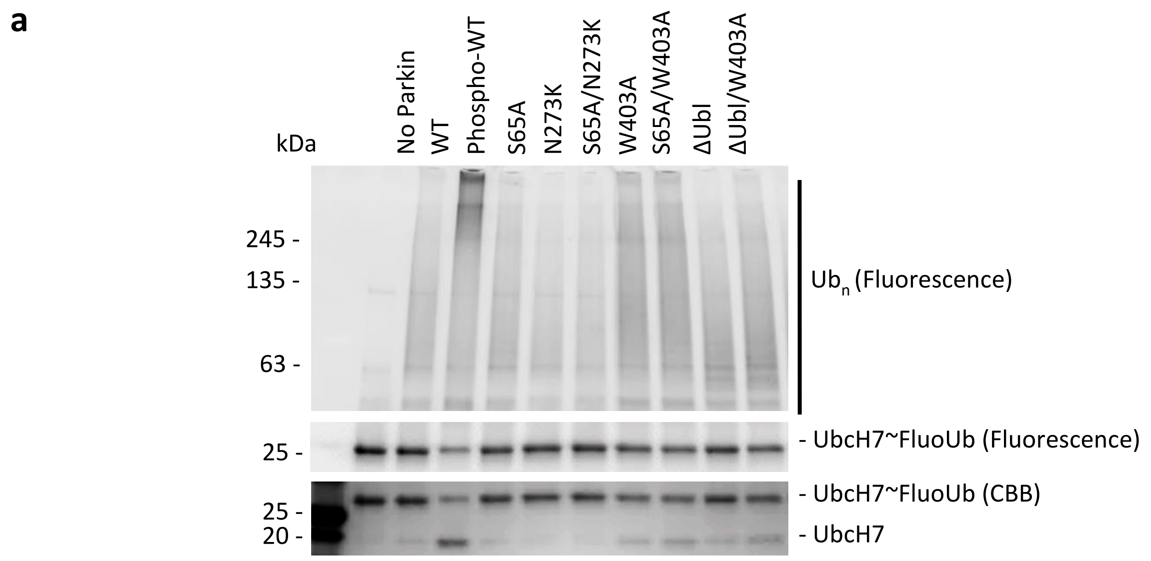

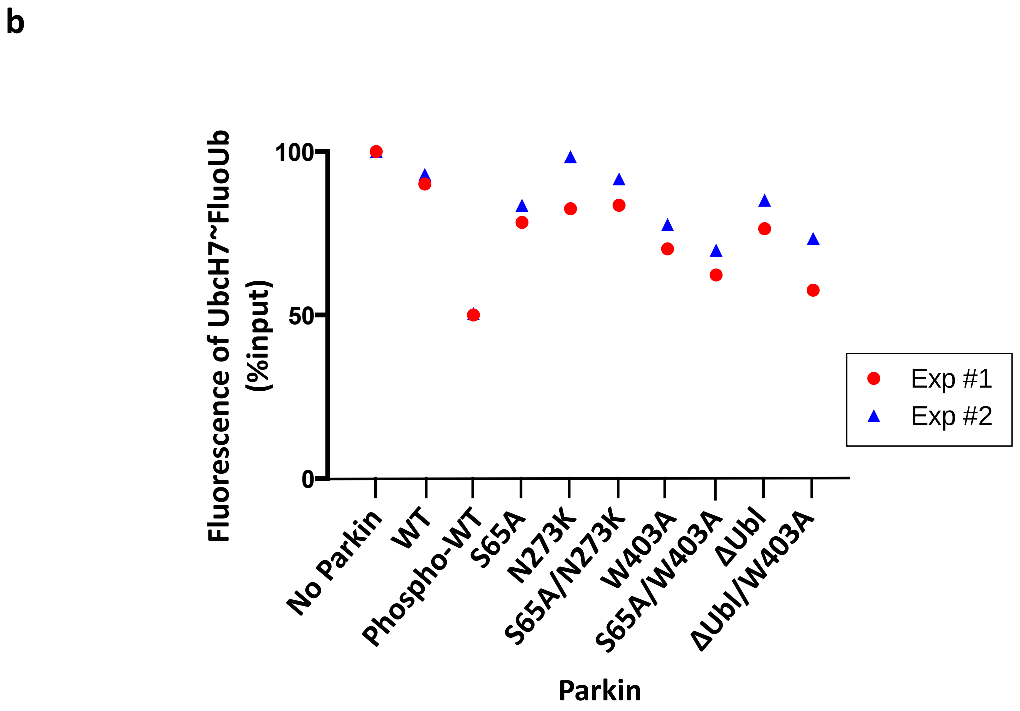

c

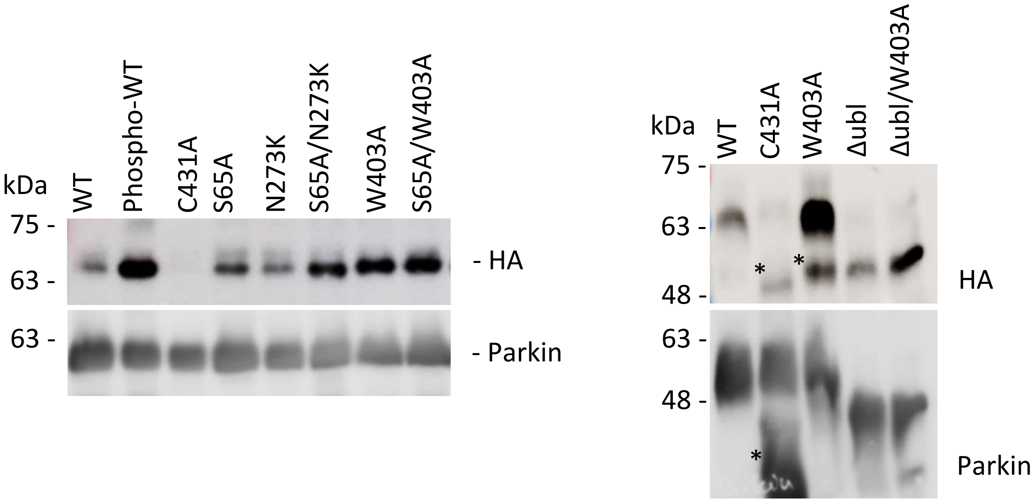

d

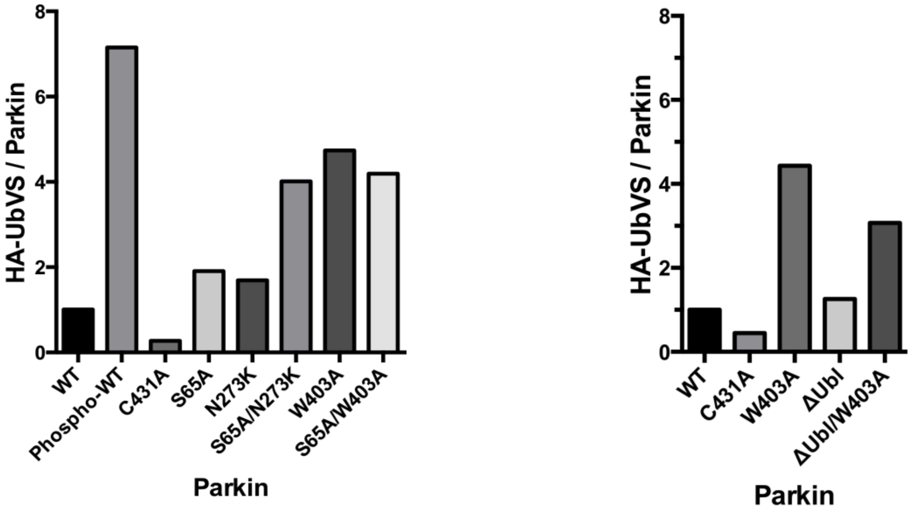

Supplementary Figure 5. *In vitro* discharging of UbcH7~FluoUb and Parkin C431 modification by Ub vinyl-sulphone (UbVS). (a) Fluorescent scan and Coomassie-stained SDS-PAGE of discharging reactions and **(b)** quantification of fluorescence strength of UbcH7~FluoUb from two independent experiments. **(c)** Western blot of UbVS modification reactions and **(d)** quantification of HA and Parkin signals (n=1). Degradation Parkin bands that expose cysteines to UbVS are marked (*).

Supplementary Figure 6

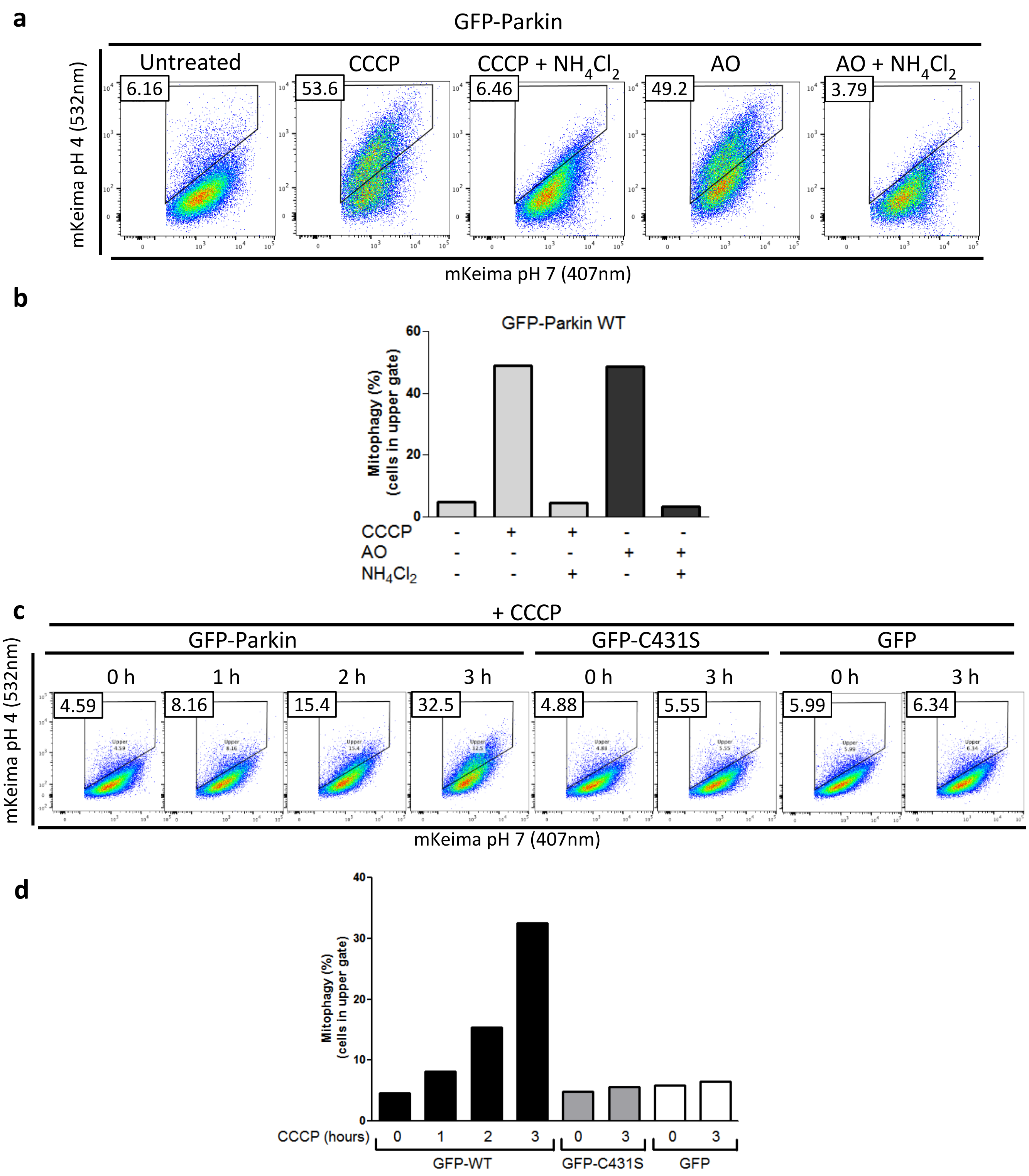

Supplementary Figure 6. **Detection of mitochondrially targeted Keima acidification after membrane depolarization. (a-b)** Fluorescence-activated cell sorting (FACS)-based analysis of U2OS cells stably expressing an ecdysone-inducible mt-Keima. Cells were induced with 10 µM ponasterone A, transfected with GFP-Parkin, and treated with 20 µM CCCP or 10 µM antimycin A and oligomycin (AO) for 4 hours. Treatment with 50 mM NH4Cl was added to neutralize the lysosomal lumen and used as a negative control. **(c-d)** Detection of mt-Keima acidification is dependent on Parkin activity. Cells expressing GFP-Parkin, C431S, or GFP alone were treated with CCCP for times indicated and analyzed by FACS (see Materials and Methods).

Supplementary Figure 7

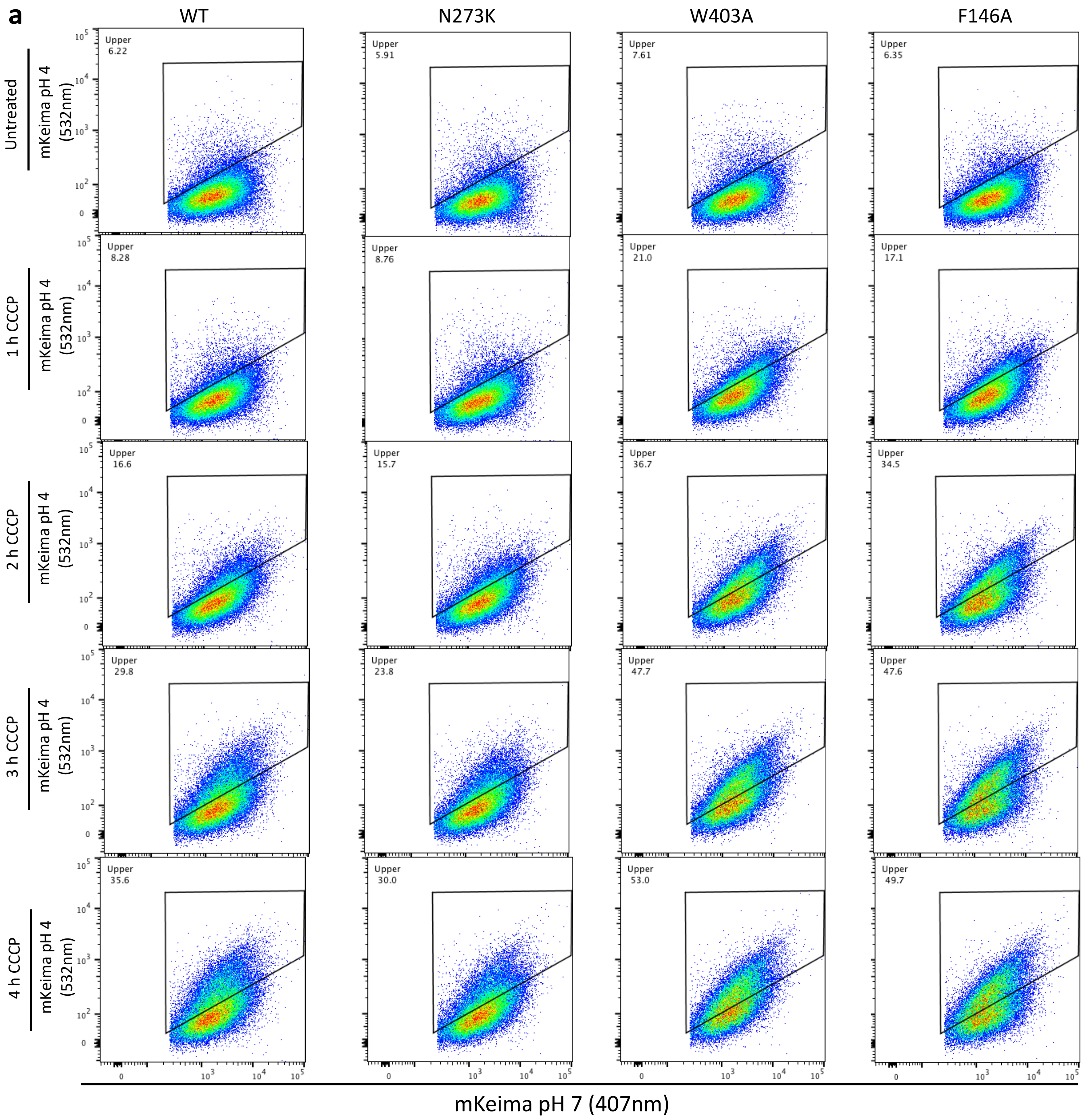

b

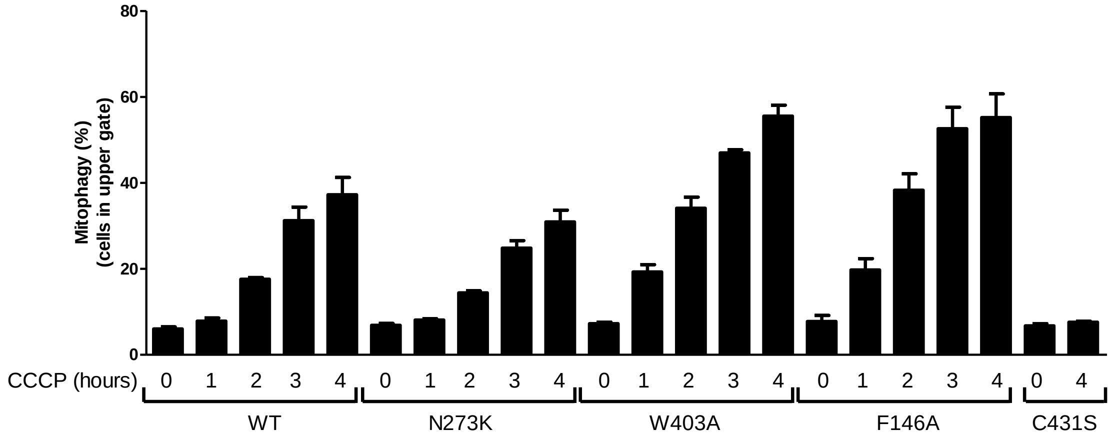

Supplementary Figure 7. **Detection of mitochondrially targeted mKeima acidification after membrane depolarization. (a)** Mitophagy was examined using a fluorescence-activated cell sorting (FACS)-based analysis of mitochondrially targeted mKeima. Cells expressing GFP-Parkin, N273K, W403A, F146A, or C431S were treated with CCCP for times indicated and analyzed by FACS. **(b)** Quantification of average percentage of mitophagy. Error bars represent SEM from 2 independent experiments.

Supplementary Figure 8

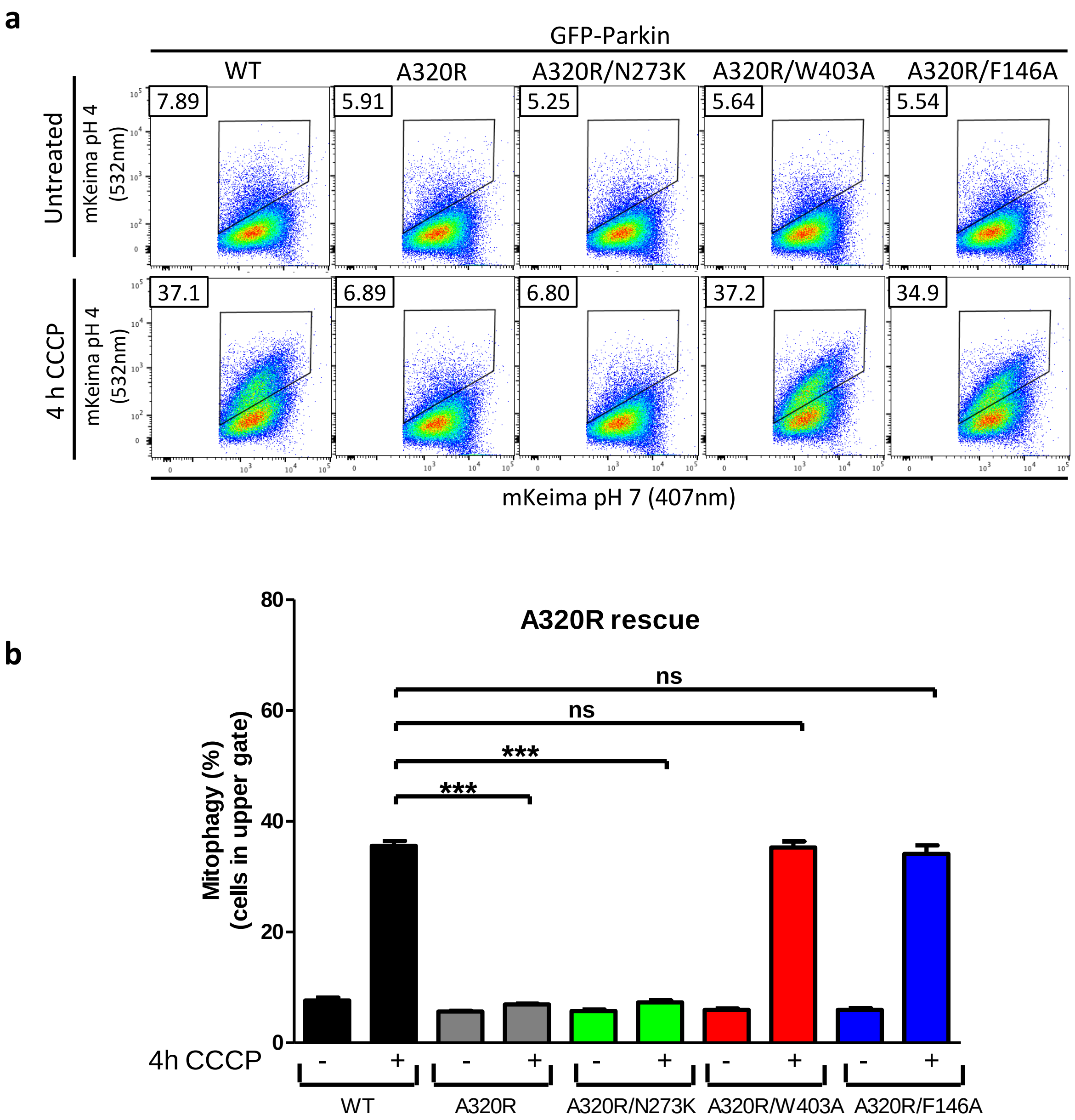

Supplementary Figure 8. Activating mutations can rescues deficits in mitophagy. (a) Representative FACS data of mt-mKeima U2OS cells expressing WT or A320R Parkin mutants untreated or treated with 20 µM CCCP for 4 hours. **(b)** Quantification of average percentage of mitophagy from three independent experiments. Error bars represents SEM. For statistical analysis, a one-way ANOVA with Tukey's post-test was performed. *** *P*<0.0001.

Supplementary Figure 9

a

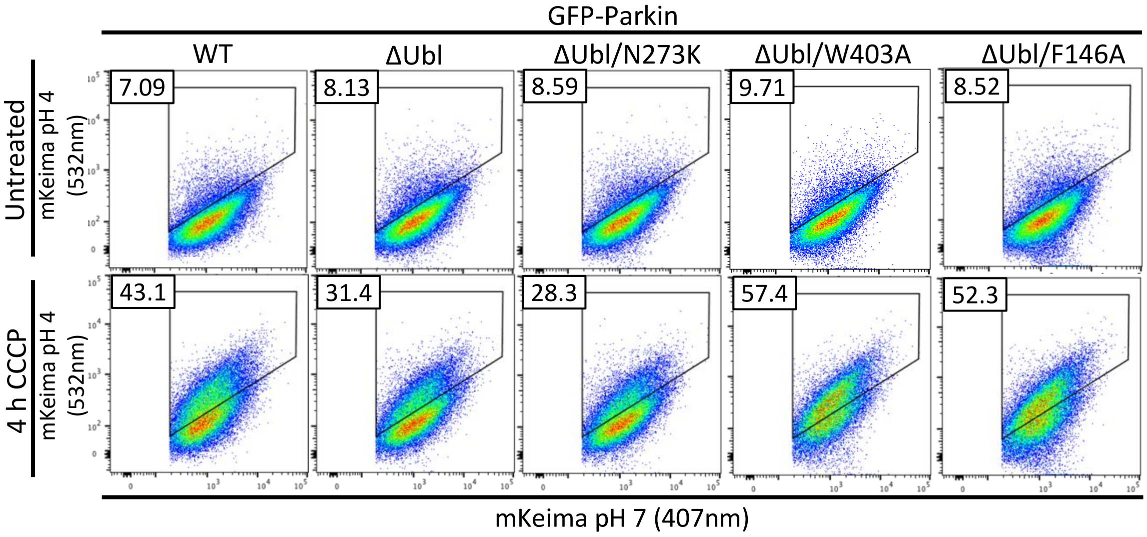

b

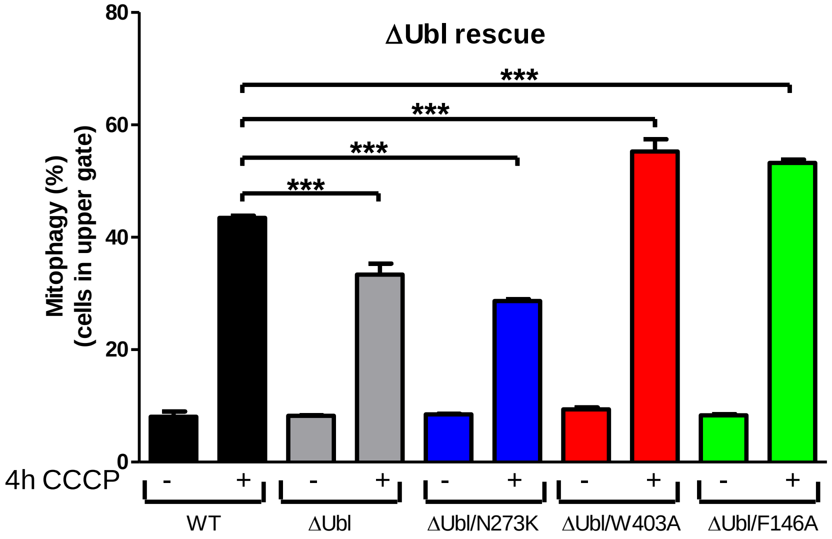

Supplementary Figure 9. Activating mutations can rescues deficits in mitophagy associated with the removal of the Ubl. (a) Representative FACS data of mt-mKeima U2OS cells expressing WT or ΔUbl Parkin mutants untreated or treated with 20 μM CCCP for 4 hours. **(b)** Quantification of average percentage of mitophagy from three independent experiments. Error bars represents SEM. For statistical analysis, a one-way ANOVA with Tukey’s post-test was performed. *** P<0.0001.

Supplementary Figure 10

a

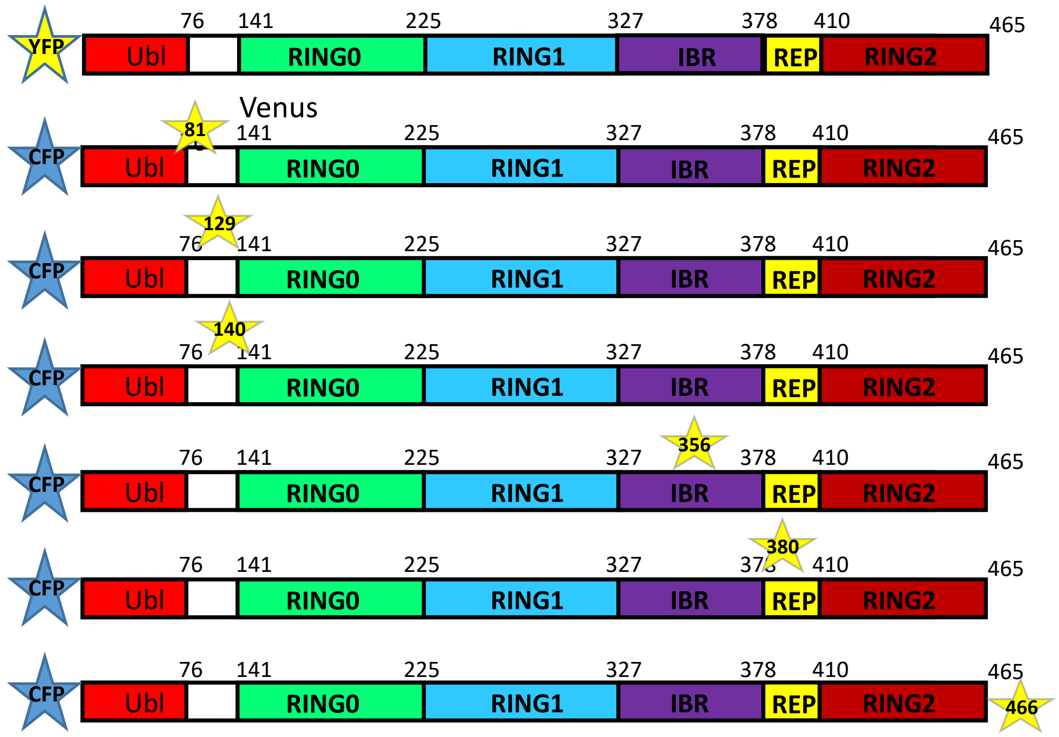

b

	Construct name	Position of Venus	Recruitment after 1 h CCCP?	Basal FRET efficiency
**i**	**YFP-Parkin**	**1 (N-term, before Ubl)**	**Yes**	**0.00**
**ii**	**FRET-81**	**81 (after Ubl)**	**Yes**	**0.25**
**iii**	**FRET-129**	**129 (in linker)**	**Yes**	**0.15**
**iv**	**FRET-140**	**140 (before RING0)**	**Less**	**0.12**
**v**	**FRET-356**	**356 (in IBR loop)**	**Less**	**0.13**
**vi**	**FRET-380**	**380 (after IBR)**	**Yes**	**0.48**
**vii**	**FRET-466**	**466 (C-terminus)**	**Much less**	**0.13**

Supplementary Figure 10. Design of Parkin FRET reporter constructs. (a) Diagram of FRET reporter constructs showing Parkin tagged with Cerulean (CFP) at its N-terminus, linker sequences and Venus (YFP) at different positions. **(b)** Summary of FRET reporter constructs from (a) comparing recruitment and FRET efficiencies for the different FRET reporters.

Supplementary Figure 11

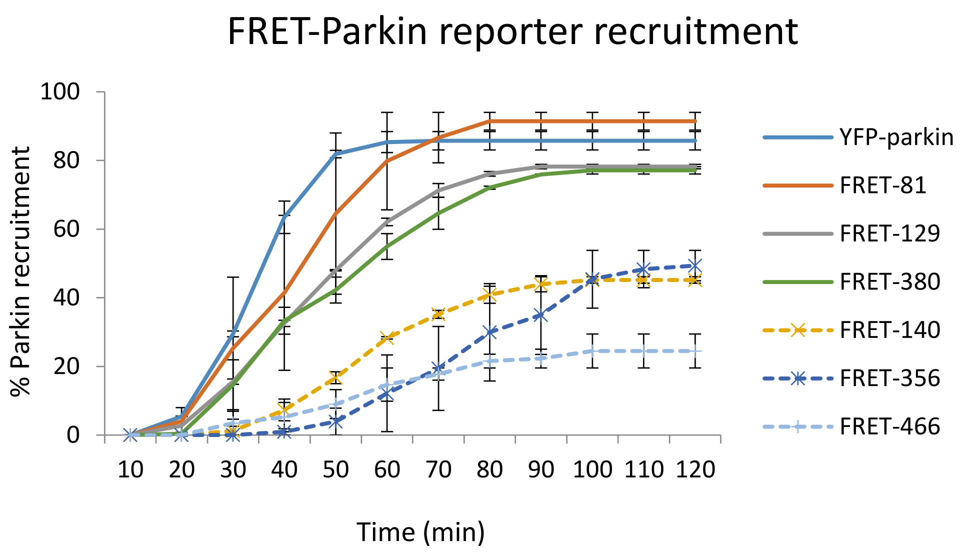

Supplementary Figure 11. Quantification of FRET-Parkin reporter recruitment to the mitochondria. Hela cells were transiently transfected with YFP-Parkin, or various FRET-Parkin reporters. The percentage of cells showing recruitment of FRET-Parkin reporters to mitochondria was determined every 10 mins over a period of 120 mins. Experiments done in duplicate with the vertical bars representing SEM.

Supplementary Figure 12

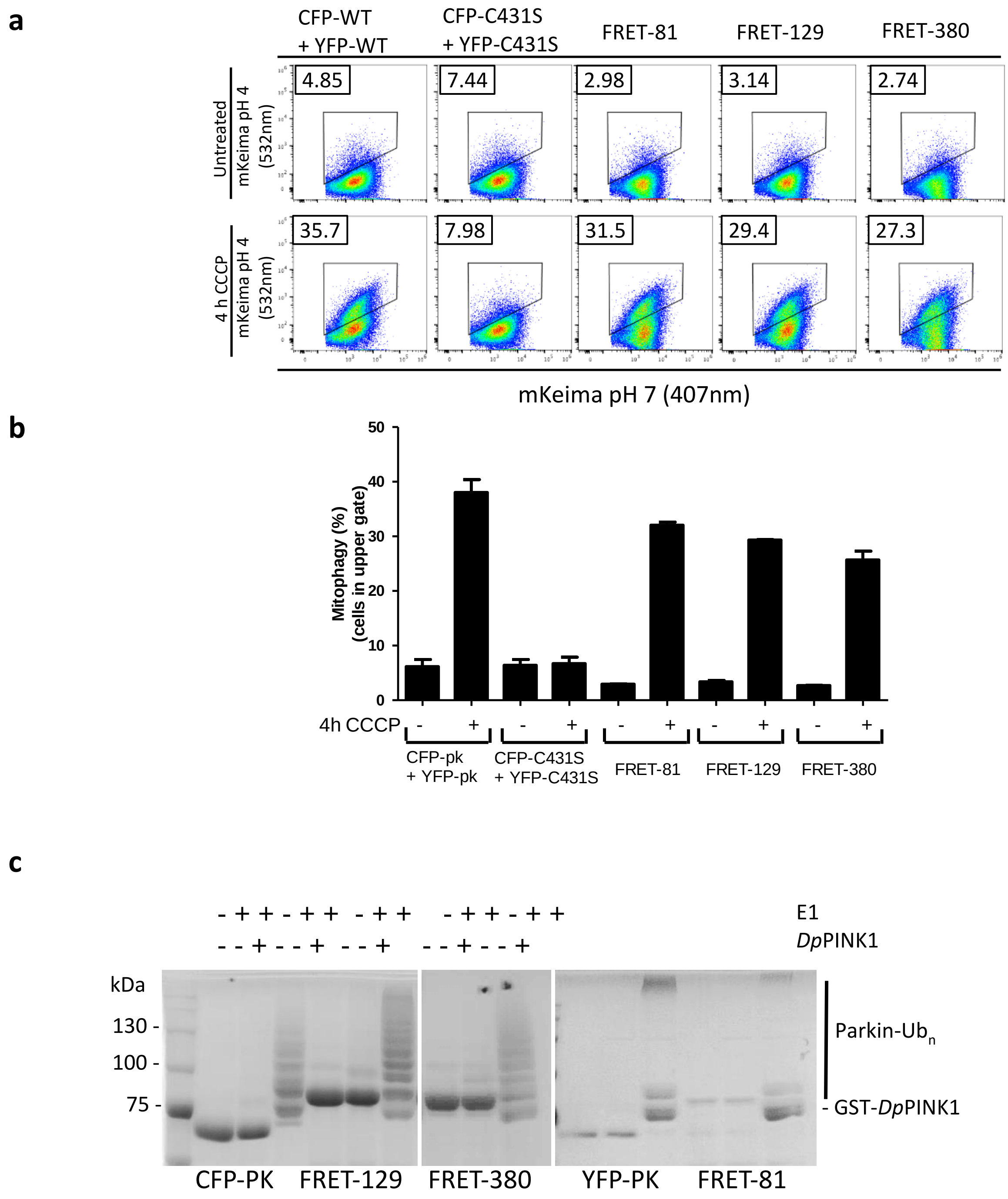

Supplementary Figure 12. **Detection of mitochondrially targeted Keima acidification after membrane depolarization.** **(a)** Mitophagy was examined using a fluorescence-activated cell sorting (FACS)-based analysis of mitochondrially targeted mKeima (mt-mKeima). Representative FACS data of mt-mKeima expressing FRET-81, FRET-129, or FRET-380 untreated or treated with CCCP for 4 hours. Cells co-transfected with CFP-Parkin WT and YFP-Parkin WT or CFP-C431S and YFP-C431S served as positive and negative controls, respectively. **(b)** Quantification of average percentage of mitophagy. Error bars represent SEM from two independent experiments. **(c)** Coomassie-stained SDS-PAGE of auto-ubiquitination reactions of purified recombinant FRET reporters. Ligase activity is monitored by the formation of higher molecular weight polyubiquitinated forms of Parkin.

Supplementary Figure 13

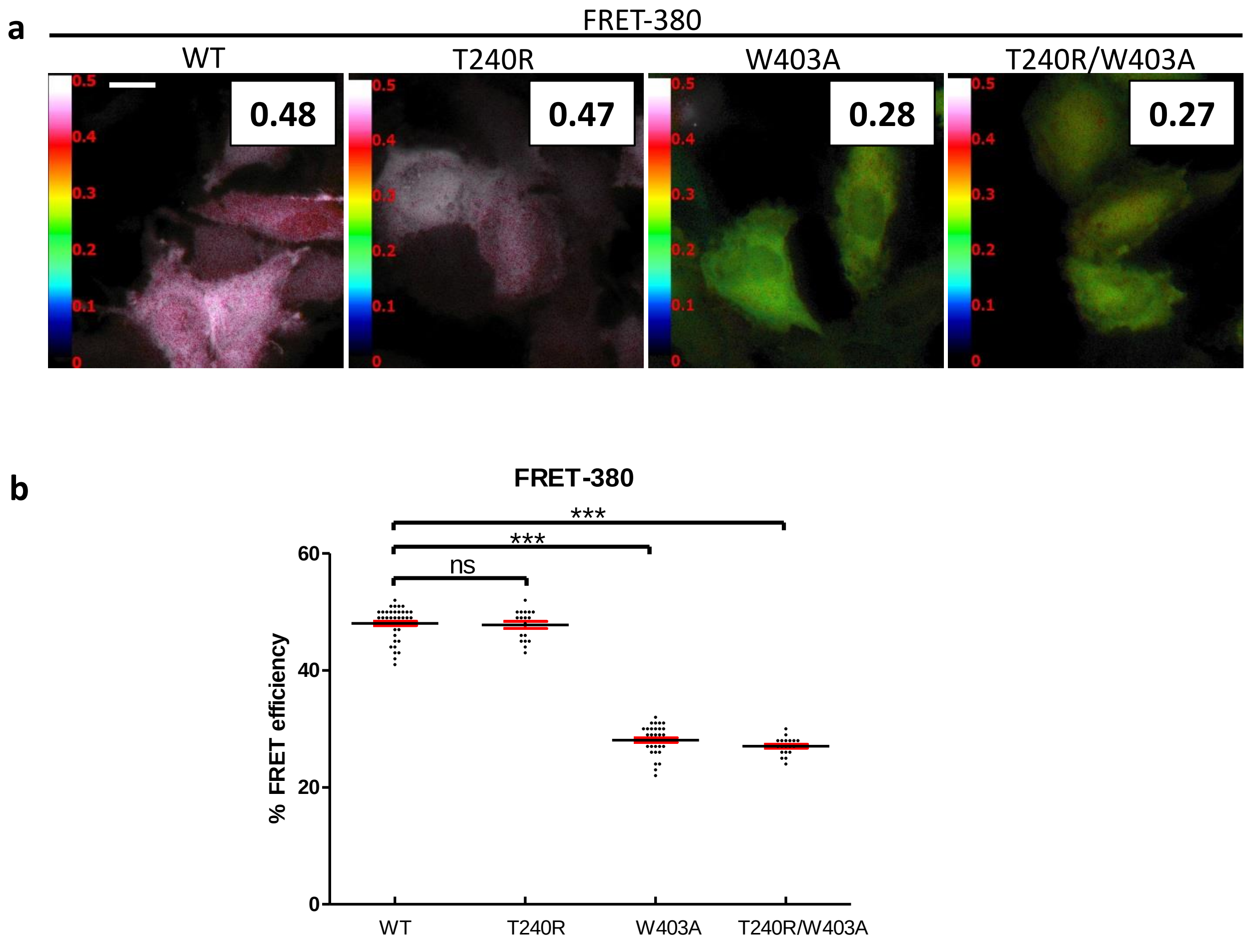

Supplementary Figure 13. Decrease in FRET observed in the W403A activating mutant in the FRET-380 reporter is not attributed to binding of E2-ubiquitin activating enzymes. (a) FRET images and **(b)** quantification of WT, E2 binding mutant T240R, or the T240R/W403A double mutant. Vertical bars represent SEM for three independent experiments. ****P* <0.0001; ns, not significant (one-way ANOVA with Tukey post-test). Scale bar: 20 µm.

Supplementary Figure 14

FRET81

ATGGTGAGCAAGGGCGAGGAGCTGTTCACCGGGGTGGTGCCCATCCTGGTCGAGCTGGACGGCGACGTAAA
CGGCCACAAGTTCAGCGTGTCCGGCGAGGGCGAGGGCGATGCCACCTACGGCAAGCTGACCCTGAAGTTCA
TCTGCACCACCGGCAAGCTGCCCGTGCCCTGGCCCACCCTCGTGACCACCCTGACCTGGGGCGTGCAGTGCT
TCGCCCGCTACCCCGACCACATGAAGCAGCACGACTTCTTCAAGTCCGCCATGCCCGAAGGCTACGTCCAG
GAGCGCACCATCTTCTTCAAGGACGACGGCAACTACAAGACCCGCGCCGAGGTGAAGTTCGAGGGCGACAC
CCTGGTGAACCGCATCGAGCTGAAGGGCATCGACTTCAAGGAGGACGGCAACATCCTGGGGCACAAGCTG
GAGTACAACGCCATCAGCGACAACGTCTATATCACCGCCGACAAGCAGAAGAACGGCATCAAGGCCAACTT
CAAGATCCGCCACAACATCGAGGACGGCAGCGTGCAGCTCGCCGACCACTACCAGCAGAACACCCCCATCG
GCGACGGCCCCGTGCTGCTGCCCGACAACCACTACCTGAGCACCCAGTCCAAGCTGAGCAAAGACCCCAAC
GAGAAGCGCGATCACATGGTCCTGCTGGAGTTCGTGACCGCCGCCGGGATCACTCTCGGCATGGACGAGCT
GTACAAGTCCGGACTCAGATCTGGAGCAATGATAGTGTTTGTCAGGTTCAACTCCAGCCATGGTTTCCCAGT
GGAGGTCGATTCTGACACCAGCATCTTCCAGCTCAAGGAGGTGGTTGCTAAGCGACAGGGGGTTCCGGCTG
ACCAGTTGCGTGTGATTTTCGCAGGGAAGGAGCTGAGGAATGACTGGACTGTGCAGAATTGTGACCTGGAT
CAGCAGAGCATTGTTCACATTGTGCAGAGACCGTGGAGAAAAGGTCAAGAAATGGTGAGCAAGGGCGAGG
AGCTGTTCACCGGGGTGGTGCCCATCCTGGTCGAGCTGGACGGCGACGTAAACGGCCACAAGTTCAGCGTG
TCCGGCGAGGGCGAGGGCGATGCCACCTACGGCAAGCTGACCCTGAAGCTCATCTGCACCACCGGCAAGCT
GCCCGTGCCCTGGCCCACCCTCGTGACCACCCTCGGCTACGGCCTGCAGTGCTTCGCCCGCTACCCCGACCA
CATGAAGCAGCACGACTTCTTCAAGTCCGCCATGCCCGAAGGCTACGTCCAGGAGCGCACCATCTTCTTCAA
GGACGACGGCAACTACAAGACCCGCGCCGAGGTGAAGTTCGAGGGCGACACCCTGGTGAACCGCATCGAG
CTGAAGGGCATCGACTTCAAGGAGGACGGCAACATCCTGGGGCACAAGCTGGAGTACAACTACAACAGCC
ACAACGTCTATATCACCGCCGACAAGCAGAAGAACGGCATCAAGGCCAACTTCAAGATCCGCCACAACATC
GAGGACGGCGGCGTGCAGCTCGCCGACCACTACCAGCAGAACACCCCCATCGGCGACGGCCCCGTGCTGCT
GCCCGACAACCACTACCTGAGCTACCAGTCCAAGCTGAGCAAAGACCCCAACGAGAAGCGCGATCACATG
GTCCTGCTGGAGTTCGTGACCGCCGCCGGGATCACTCTCGGCATGGACGAGCTGTACAAGAATGCAACTGG
AGGCGACGACCCCAGAAACGCGGCGGGAGGCTGTGAGCGGGAGCCCCAGAGCTTGACTCGGGTGGACCTC
AGCAGCTCAGTCCTCCCAGGAGACTCTGTGGGGCTGGCTGTCATTCTGCACACTGACAGCAGGAAGGACTC
ACCACCAGCTGGAAGTCCAGCAGGTAGATCAATCTACAACAGCTTTTATGTGTATTGCAAAGGCCCCTGTCA
AAGAGTGCAGCCGGGAAAACTCAGGGTACAGTGCAGCACCTGCAGGCAGGCAACGCTCACCTTGACCCAG
GGTCCATCTTGCTGGGATGATGTTTTAATTCCAAACCGGATGAGTGGTGAATGCCAATCCCCACACTGCCCT
GGGACTAGTGCAGAATTTTTCTTTAAATGTGGAGCACACCCCACCTCTGACAAGGAAACATCAGTAGCTTTG
CACCTGATCGCAACAAATAGTCGGAACATCACTTGCATTACGTGCACAGACGTCAGGAGCCCCGTCCTGGT
TTTCCAGTGCAACTCCCGCCACGTGATTTGCTTAGACTGTTTCCACTTATACTGTGTGACAAGACTCAATGAT
CGGCAGTTTGTTCACGACCCTCAACTTGGCTACTCCCTGCCTTGTGTGGCTGGCTGTCCCAACTCCTTGATTA
AAGAGCTCCATCACTTCAGGATTCTGGGAGAAGAGCAGTACAACCGGTACCAGCAGTATGGTGCAGAGGA
GTGTGTCCTGCAGATGGGGGGCGTGTTATGCCCCCGCCCTGGCTGTGGAGCGGGGCTGCTGCCGGAGCCTG
ACCAGAGGAAAGTCACCTGCGAAGGGGGCAATGGCCTGGGCTGTGGGTTTGCCTTCTGCCGGGAATGTAAA
GAAGCGTACCATGAAGGGGAGTGCAGTGCCGTATTTGAAGCCTCAGGAACAACTACTCAGGCCTACAGAGT
CGATGAAAGAGCCGCCGAGCAGGCTCGTTGGGAAGCAGCCTCCAAAGAAACCATCAAGAAAACCACCAAG
CCCTGTCCCCGCTGCCATGTACCAGTGGAAAAAAATGGAGGCTGCATGCACATGAAGTGTCCGCAGCCCCA
GTGCAGGCTCGAGTGGTGCTGGAACTGTGGCTGCGAGTGGAACCGCGTCTGCATGGGGGACCACTGGTTCG
ACGTGTAG

Supplementary Figure 14 (continued)

FRET129

ATGGTGAGCAAGGGCGAGGAGCTGTTCACCGGGGTGGTGCCCATCCTGGTCGAGCTGGACGGCGACGTAAA
CGGCCACAAGTTCAGCGTGTCCGGCGAGGGCGAGGGCGATGCCACCTACGGCAAGCTGACCCTGAAGTTCA
TCTGCACCACCGGCAAGCTGCCCGTGCCCTGGCCCACCCTCGTGACCACCCTGACCTGGGGCGTGCAGTGCT
TCGCCCGCTACCCCGACCACATGAAGCAGCACGACTTCTTCAAGTCCGCCATGCCCGAAGGCTACGTCCAG
GAGCGCACCATCTTCTTCAAGGACGACGGCAACTACAAGACCCGCGCCGAGGTGAAGTTCGAGGGCGACAC
CCTGGTGAACCGCATCGAGCTGAAGGGCATCGACTTCAAGGAGGACGGCAACATCCTGGGGCACAAGCTG
GAGTACAACGCCATCAGCGACAACGTCTATATCACCGCCGACAAGCAGAAGAACGGCATCAAGGCCAACTT
CAAGATCCGCCACAACATCGAGGACGGCAGCGTGCAGCTCGCCGACCACTACCAGCAGAACACCCCCATCG
GCGACGGCCCCGTGCTGCTGCCCGACAACCACTACCTGAGCACCCAGTCCAAGCTGAGCAAAGACCCCAAC
GAGAAGCGCGATCACATGGTCCTGCTGGAGTTCGTGACCGCCGCCGGGATCACTCTCGGCATGGACGAGCT
GTACAAGTCCGGACTCAGATCTGGAGCAATGATAGTGTTTGTCAGGTTCAACTCCAGCCATGGTTTCCCAGT
GGAGGTCGATTCTGACACCAGCATCTTCCAGCTCAAGGAGGTGGTTGCTAAGCGACAGGGGGTTCCGGCTG
ACCAGTTGCGTGTGATTTTCGCAGGGAAGGAGCTGAGGAATGACTGGACTGTGCAGAATTGTGACCTGGAT
CAGCAGAGCATTGTTCACATTGTGCAGAGACCGTGGAGAAAAGGTCAAGAAATGAATGCAACTGGAGGCG
ACGACCCCAGAAACGCGGCGGGAGGCTGTGAGCGGGAGCCCCAGAGCTTGACTCGGGTGGACCTCAGCAG
CTCAGTCCTCCCAGGAGACTCTGTGGGGCTGGCTGTCATTCTGCACACTGACAGCAGGATGGTGAGCAAGG
GCGAGGAGCTGTTCACCGGGGTGGTGCCCATCCTGGTCGAGCTGGACGGCGACGTAAACGGCCACAAGTTC
AGCGTGTCCGGCGAGGGCGAGGGCGATGCCACCTACGGCAAGCTGACCCTGAAGCTCATCTGCACCACCGG
CAAGCTGCCCGTGCCCTGGCCCACCCTCGTGACCACCCTCGGCTACGGCCTGCAGTGCTTCGCCCGCTACCC
CGACCACATGAAGCAGCACGACTTCTTCAAGTCCGCCATGCCCGAAGGCTACGTCCAGGAGCGCACCATCT
TCTTCAAGGACGACGGCAACTACAAGACCCGCGCCGAGGTGAAGTTCGAGGGCGACACCCTGGTGAACCGC
ATCGAGCTGAAGGGCATCGACTTCAAGGAGGACGGCAACATCCTGGGGCACAAGCTGGAGTACAACTACA
ACAGCCACAACGTCTATATCACCGCCGACAAGCAGAAGAACGGCATCAAGGCCAACTTCAAGATCCGCCAC
AACATCGAGGACGGCGGCGTGCAGCTCGCCGACCACTACCAGCAGAACACCCCCATCGGCGACGGCCCCGT
GCTGCTGCCCGACAACCACTACCTGAGCTACCAGTCCAAGCTGAGCAAAGACCCCAACGAGAAGCGCGATC
ACATGGTCCTGCTGGAGTTCGTGACCGCCGCCGGGATCACTCTCGGCATGGACGAGCTGTACAAGAAGGAC
TCACCACCAGCTGGAAGTCCAGCAGGTAGATCAATCTACAACAGCTTTTATGTGTATTGCAAAGGCCCCTGT
CAAAGAGTGCAGCCGGGAAAACTCAGGGTACAGTGCAGCACCTGCAGGCAGGCAACGCTCACCTTGACCC
AGGGTCCATCTTGCTGGGATGATGTTTTAATTCCAAACCGGATGAGTGGTGAATGCCAATCCCCACACTGCC
CTGGGACTAGTGCAGAATTTTTCTTTAAATGTGGAGCACACCCCACCTCTGACAAGGAAACATCAGTAGCTT
TGCACCTGATCGCAACAAATAGTCGGAACATCACTTGCATTACGTGCACAGACGTCAGGAGCCCCGTCCTG
GTTTTCCAGTGCAACTCCCGCCACGTGATTTGCTTAGACTGTTTCCACTTATACTGTGTGACAAGACTCAATG
ATCGGCAGTTTGTTCACGACCCTCAACTTGGCTACTCCCTGCCTTGTGTGGCTGGCTGTCCCAACTCCTTGAT
TAAAGAGCTCCATCACTTCAGGATTCTGGGAGAAGAGCAGTACAACCGGTACCAGCAGTATGGTGCAGAGG
AGTGTGTCCTGCAGATGGGGGGCGTGTTATGCCCCCGCCCTGGCTGTGGAGCGGGGCTGCTGCCGGAGCCT
GACCAGAGGAAAGTCACCTGCGAAGGGGGCAATGGCCTGGGCTGTGGGTTTGCCTTCTGCCGGGAATGTAA
AGAAGCGTACCATGAAGGGGAGTGCAGTGCCGTATTTGAAGCCTCAGGAACAACTACTCAGGCCTACAGAG
TCGATGAAAGAGCCGCCGAGCAGGCTCGTTGGGAAGCAGCCTCCAAAGAAACCATCAAGAAAACCACCAA
GCCCTGTCCCCGCTGCCATGTACCAGTGGAAAAAAATGGAGGCTGCATGCACATGAAGTGTCCGCAGCCCC
AGTGCAGGCTCGAGTGGTGCTGGAACTGTGGCTGCGAGTGGAACCGCGTCTGCATGGGGGACCACTGGTTC
GACGTGTAG

Supplementary Figure 14 (continued)

FRET140

ATGGTGAGCAAGGGCGAGGAGCTGTTCACCGGGGTGGTGCCCATCCTGGTCGAGCTGGACGGCGACGTAAA
CGGCCACAAGTTCAGCGTGTCCGGCGAGGGCGAGGGCGATGCCACCTACGGCAAGCTGACCCTGAAGTTCA
TCTGCACCACCGGCAAGCTGCCCGTGCCCTGGCCCACCCTCGTGACCACCCTGACCTGGGGCGTGCAGTGCT
TCGCCCGCTACCCCGACCACATGAAGCAGCACGACTTCTTCAAGTCCGCCATGCCCGAAGGCTACGTCCAG
GAGCGCACCATCTTCTTCAAGGACGACGGCAACTACAAGACCCGCGCCGAGGTGAAGTTCGAGGGCGACAC
CCTGGTGAACCGCATCGAGCTGAAGGGCATCGACTTCAAGGAGGACGGCAACATCCTGGGGCACAAGCTG
GAGTACAACGCCATCAGCGACAACGTCTATATCACCGCCGACAAGCAGAAGAACGGCATCAAGGCCAACTT
CAAGATCCGCCACAACATCGAGGACGGCAGCGTGCAGCTCGCCGACCACTACCAGCAGAACACCCCCATCG
GCGACGGCCCCGTGCTGCTGCCCGACAACCACTACCTGAGCACCCAGTCCAAGCTGAGCAAAGACCCCAAC
GAGAAGCGCGATCACATGGTCCTGCTGGAGTTCGTGACCGCCGCCGGGATCACTCTCGGCATGGACGAGCT
GTACAAGTCCGGACTCAGATCTGGAGCAATGATAGTGTTTGTCAGGTTCAACTCCAGCCATGGTTTCCCAGT
GGAGGTCGATTCTGACACCAGCATCTTCCAGCTCAAGGAGGTGGTTGCTAAGCGACAGGGGGTTCCGGCTG
ACCAGTTGCGTGTGATTTTCGCAGGGAAGGAGCTGAGGAATGACTGGACTGTGCAGAATTGTGACCTGGAT
CAGCAGAGCATTGTTCACATTGTGCAGAGACCGTGGAGAAAAGGTCAAGAAATGAATGCAACTGGAGGCG
ACGACCCCAGAAACGCGGCGGGAGGCTGTGAGCGGGAGCCCCAGAGCTTGACTCGGGTGGACCTCAGCAG
CTCAGTCCTCCCAGGAGACTCTGTGGGGCTGGCTGTCATTCTGCACACTGACAGCAGGAAGGACTCACCAC
CAGCTGGAAGTCCAGCAGGTATGGTGAGCAAGGGCGAGGAGCTGTTCACCGGGGTGGTGCCCATCCTGGTC
GAGCTGGACGGCGACGTAAACGGCCACAAGTTCAGCGTGTCCGGCGAGGGCGAGGGCGATGCCACCTACG
GCAAGCTGACCCTGAAGCTCATCTGCACCACCGGCAAGCTGCCCGTGCCCTGGCCCACCCTCGTGACCACCC
TCGGCTACGGCCTGCAGTGCTTCGCCCGCTACCCCGACCACATGAAGCAGCACGACTTCTTCAAGTCCGCCA
TGCCCGAAGGCTACGTCCAGGAGCGCACCATCTTCTTCAAGGACGACGGCAACTACAAGACCCGCGCCGAG
GTGAAGTTCGAGGGCGACACCCTGGTGAACCGCATCGAGCTGAAGGGCATCGACTTCAAGGAGGACGGCA
ACATCCTGGGGCACAAGCTGGAGTACAACTACAACAGCCACAACGTCTATATCACCGCCGACAAGCAGAAG
AACGGCATCAAGGCCAACTTCAAGATCCGCCACAACATCGAGGACGGCGGCGTGCAGCTCGCCGACCACTA
CCAGCAGAACACCCCCATCGGCGACGGCCCCGTGCTGCTGCCCGACAACCACTACCTGAGCTACCAGTCCA
AGCTGAGCAAAGACCCCAACGAGAAGCGCGATCACATGGTCCTGCTGGAGTTCGTGACCGCCGCCGGGATC
ACTCTCGGCATGGACGAGCTGTACAAGAGATCAATCTACAACAGCTTTTATGTGTATTGCAAAGGCCCCTGT
CAAAGAGTGCAGCCGGGAAAACTCAGGGTACAGTGCAGCACCTGCAGGCAGGCAACGCTCACCTTGACCC
AGGGTCCATCTTGCTGGGATGATGTTTTAATTCCAAACCGGATGAGTGGTGAATGCCAATCCCCACACTGCC
CTGGGACTAGTGCAGAATTTTTCTTTAAATGTGGAGCACACCCCACCTCTGACAAGGAAACATCAGTAGCTT
TGCACCTGATCGCAACAAATAGTCGGAACATCACTTGCATTACGTGCACAGACGTCAGGAGCCCCGTCCTG
GTTTTCCAGTGCAACTCCCGCCACGTGATTTGCTTAGACTGTTTCCACTTATACTGTGTGACAAGACTCAATG
ATCGGCAGTTTGTTCACGACCCTCAACTTGGCTACTCCCTGCCTTGTGTGGCTGGCTGTCCCAACTCCTTGAT
TAAAGAGCTCCATCACTTCAGGATTCTGGGAGAAGAGCAGTACAACCGGTACCAGCAGTATGGTGCAGAGG
AGTGTGTCCTGCAGATGGGGGGCGTGTTATGCCCCCGCCCTGGCTGTGGAGCGGGGCTGCTGCCGGAGCCT
GACCAGAGGAAAGTCACCTGCGAAGGGGGCAATGGCCTGGGCTGTGGGTTTGCCTTCTGCCGGGAATGTAA
AGAAGCGTACCATGAAGGGGAGTGCAGTGCCGTATTTGAAGCCTCAGGAACAACTACTCAGGCCTACAGAG
TCGATGAAAGAGCCGCCGAGCAGGCTCGTTGGGAAGCAGCCTCCAAAGAAACCATCAAGAAAACCACCAA
GCCCTGTCCCCGCTGCCATGTACCAGTGGAAAAAAATGGAGGCTGCATGCACATGAAGTGTCCGCAGCCCC
AGTGCAGGCTCGAGTGGTGCTGGAACTGTGGCTGCGAGTGGAACCGCGTCTGCATGGGGGACCACTGGTTC
GACGTGTAG

Supplementary Figure 14 (continued)

FRET356

ATGGTGAGCAAGGGCGAGGAGCTGTTCACCGGGGTGGTGCCCATCCTGGTCGAGCTGGACGGCGACGTAAA
CGGCCACAAGTTCAGCGTGTCCGGCGAGGGCGAGGGCGATGCCACCTACGGCAAGCTGACCCTGAAGTTCA
TCTGCACCACCGGCAAGCTGCCCGTGCCCTGGCCCACCCTCGTGACCACCCTGACCTGGGGCGTGCAGTGCT
TCGCCCGCTACCCCGACCACATGAAGCAGCACGACTTCTTCAAGTCCGCCATGCCCGAAGGCTACGTCCAG
GAGCGCACCATCTTCTTCAAGGACGACGGCAACTACAAGACCCGCGCCGAGGTGAAGTTCGAGGGCGACAC
CCTGGTGAACCGCATCGAGCTGAAGGGCATCGACTTCAAGGAGGACGGCAACATCCTGGGGCACAAGCTG
GAGTACAACGCCATCAGCGACAACGTCTATATCACCGCCGACAAGCAGAAGAACGGCATCAAGGCCAACTT
CAAGATCCGCCACAACATCGAGGACGGCAGCGTGCAGCTCGCCGACCACTACCAGCAGAACACCCCCATCG
GCGACGGCCCCGTGCTGCTGCCCGACAACCACTACCTGAGCACCCAGTCCAAGCTGAGCAAAGACCCCAAC
GAGAAGCGCGATCACATGGTCCTGCTGGAGTTCGTGACCGCCGCCGGGATCACTCTCGGCATGGACGAGCT
GTACAAGTCCGGACTCAGATCTGGAGCAATGATAGTGTTTGTCAGGTTCAACTCCAGCCATGGTTTCCCAGT
GGAGGTCGATTCTGACACCAGCATCTTCCAGCTCAAGGAGGTGGTTGCTAAGCGACAGGGGGTTCCGGCTG
ACCAGTTGCGTGTGATTTTCGCAGGGAAGGAGCTGAGGAATGACTGGACTGTGCAGAATTGTGACCTGGAT
CAGCAGAGCATTGTTCACATTGTGCAGAGACCGTGGAGAAAAGGTCAAGAAATGAATGCAACTGGAGGCG
ACGACCCCAGAAACGCGGCGGGAGGCTGTGAGCGGGAGCCCCAGAGCTTGACTCGGGTGGACCTCAGCAG
CTCAGTCCTCCCAGGAGACTCTGTGGGGCTGGCTGTCATTCTGCACACTGACAGCAGGAAGGACTCACCAC
CAGCTGGAAGTCCAGCAGGTAGATCAATCTACAACAGCTTTTATGTGTATTGCAAAGGCCCCTGTCAAAGA
GTGCAGCCGGGAAAACTCAGGGTACAGTGCAGCACCTGCAGGCAGGCAACGCTCACCTTGACCCAGGGTCC
ATCTTGCTGGGATGATGTTTTAATTCCAAACCGGATGAGTGGTGAATGCCAATCCCCACACTGCCCTGGGAC
TAGTGCAGAATTTTTCTTTAAATGTGGAGCACACCCCACCTCTGACAAGGAAACATCAGTAGCTTTGCACCT
GATCGCAACAAATAGTCGGAACATCACTTGCATTACGTGCACAGACGTCAGGAGCCCCGTCCTGGTTTTCCA
GTGCAACTCCCGCCACGTGATTTGCTTAGACTGTTTCCACTTATACTGTGTGACAAGACTCAATGATCGGCA
GTTTGTTCACGACCCTCAACTTGGCTACTCCCTGCCTTGTGTGGCTGGCTGTCCCAACTCCTTGATTAAAGAG
CTCCATCACTTCAGGATTCTGGGAGAAGAGCAGTACAACCGGTACCAGCAGTATGGTGCAGAGGAGTGTGT
CCTGCAGATGGGGGGCGTGTTATGCCCCCGCCCTGGCTGTGGAGCGGGGCTGCTGCCGGAGCCTGACCAGA
GGAAAGTCACCTGCGAAGGGGGCATGGTGAGCAAGGGCGAGGAGCTGTTCACCGGGGTGGTGCCCATCCT
GGTCGAGCTGGACGGCGACGTAAACGGCCACAAGTTCAGCGTGTCCGGCGAGGGCGAGGGCGATGCCACC
TACGGCAAGCTGACCCTGAAGCTCATCTGCACCACCGGCAAGCTGCCCGTGCCCTGGCCCACCCTCGTGACC
ACCCTCGGCTACGGCCTGCAGTGCTTCGCCCGCTACCCCGACCACATGAAGCAGCACGACTTCTTCAAGTCC
GCCATGCCCGAAGGCTACGTCCAGGAGCGCACCATCTTCTTCAAGGACGACGGCAACTACAAGACCCGCGC
CGAGGTGAAGTTCGAGGGCGACACCCTGGTGAACCGCATCGAGCTGAAGGGCATCGACTTCAAGGAGGAC
GGCAACATCCTGGGGCACAAGCTGGAGTACAACTACAACAGCCACAACGTCTATATCACCGCCGACAAGCA
GAAGAACGGCATCAAGGCCAACTTCAAGATCCGCCACAACATCGAGGACGGCGGCGTGCAGCTCGCCGAC
CACTACCAGCAGAACACCCCCATCGGCGACGGCCCCGTGCTGCTGCCCGACAACCACTACCTGAGCTACCA
GTCCAAGCTGAGCAAAGACCCCAACGAGAAGCGCGATCACATGGTCCTGCTGGAGTTCGTGACCGCCGCCG
GGATCACTCTCGGCATGGACGAGCTGTACAAGAATGGCCTGGGCTGTGGGTTTGCCTTCTGCCGGGAATGT
AAAGAAGCGTACCATGAAGGGGAGTGCAGTGCCGTATTTGAAGCCTCAGGAACAACTACTCAGGCCTACAG
AGTCGATGAAAGAGCCGCCGAGCAGGCTCGTTGGGAAGCAGCCTCCAAAGAAACCATCAAGAAAACCACC
AAGCCCTGTCCCCGCTGCCATGTACCAGTGGAAAAAAATGGAGGCTGCATGCACATGAAGTGTCCGCAGCC
CCAGTGCAGGCTCGAGTGGTGCTGGAACTGTGGCTGCGAGTGGAACCGCGTCTGCATGGGGGACCACTGGT
TCGACGTGTAG

Supplementary Figure 14 (continued)

FRET380

ATGGTGAGCAAGGGCGAGGAGCTGTTCACCGGGGTGGTGCCCATCCTGGTCGAGCTGGACGGCGACGTAAA
CGGCCACAAGTTCAGCGTGTCCGGCGAGGGCGAGGGCGATGCCACCTACGGCAAGCTGACCCTGAAGTTCA
TCTGCACCACCGGCAAGCTGCCCGTGCCCTGGCCCACCCTCGTGACCACCCTGACCTGGGGCGTGCAGTGCT
TCGCCCGCTACCCCGACCACATGAAGCAGCACGACTTCTTCAAGTCCGCCATGCCCGAAGGCTACGTCCAG
GAGCGCACCATCTTCTTCAAGGACGACGGCAACTACAAGACCCGCGCCGAGGTGAAGTTCGAGGGCGACAC
CCTGGTGAACCGCATCGAGCTGAAGGGCATCGACTTCAAGGAGGACGGCAACATCCTGGGGCACAAGCTG
GAGTACAACGCCATCAGCGACAACGTCTATATCACCGCCGACAAGCAGAAGAACGGCATCAAGGCCAACTT
CAAGATCCGCCACAACATCGAGGACGGCAGCGTGCAGCTCGCCGACCACTACCAGCAGAACACCCCCATCG
GCGACGGCCCCGTGCTGCTGCCCGACAACCACTACCTGAGCACCCAGTCCAAGCTGAGCAAAGACCCCAAC
GAGAAGCGCGATCACATGGTCCTGCTGGAGTTCGTGACCGCCGCCGGGATCACTCTCGGCATGGACGAGCT
GTACAAGTCCGGACTCAGATCTGGAGCAATGATAGTGTTTGTCAGGTTCAACTCCAGCCATGGTTTCCCAGT
GGAGGTCGATTCTGACACCAGCATCTTCCAGCTCAAGGAGGTGGTTGCTAAGCGACAGGGGGTTCCGGCTG
ACCAGTTGCGTGTGATTTTCGCAGGGAAGGAGCTGAGGAATGACTGGACTGTGCAGAATTGTGACCTGGAT
CAGCAGAGCATTGTTCACATTGTGCAGAGACCGTGGAGAAAAGGTCAAGAAATGAATGCAACTGGAGGCG
ACGACCCCAGAAACGCGGCGGGAGGCTGTGAGCGGGAGCCCCAGAGCTTGACTCGGGTGGACCTCAGCAG
CTCAGTCCTCCCAGGAGACTCTGTGGGGCTGGCTGTCATTCTGCACACTGACAGCAGGAAGGACTCACCAC
CAGCTGGAAGTCCAGCAGGTAGATCAATCTACAACAGCTTTTATGTGTATTGCAAAGGCCCCTGTCAAAGA
GTGCAGCCGGGAAAACTCAGGGTACAGTGCAGCACCTGCAGGCAGGCAACGCTCACCTTGACCCAGGGTCC
ATCTTGCTGGGATGATGTTTTAATTCCAAACCGGATGAGTGGTGAATGCCAATCCCCACACTGCCCTGGGAC
TAGTGCAGAATTTTTCTTTAAATGTGGAGCACACCCCACCTCTGACAAGGAAACATCAGTAGCTTTGCACCT
GATCGCAACAAATAGTCGGAACATCACTTGCATTACGTGCACAGACGTCAGGAGCCCCGTCCTGGTTTTCCA
GTGCAACTCCCGCCACGTGATTTGCTTAGACTGTTTCCACTTATACTGTGTGACAAGACTCAATGATCGGCA
GTTTGTTCACGACCCTCAACTTGGCTACTCCCTGCCTTGTGTGGCTGGCTGTCCCAACTCCTTGATTAAAGAG
CTCCATCACTTCAGGATTCTGGGAGAAGAGCAGTACAACCGGTACCAGCAGTATGGTGCAGAGGAGTGTGT
CCTGCAGATGGGGGGCGTGTTATGCCCCCGCCCTGGCTGTGGAGCGGGGCTGCTGCCGGAGCCTGACCAGA
GGAAAGTCACCTGCGAAGGGGGCAATGGCCTGGGCTGTGGGTTTGCCTTCTGCCGGGAATGTAAAGAAGCG
TACCATGAAGGGGAGTGCAGTGCCATGGTGAGCAAGGGCGAGGAGCTGTTCACCGGGGTGGTGCCCATCCT
GGTCGAGCTGGACGGCGACGTAAACGGCCACAAGTTCAGCGTGTCCGGCGAGGGCGAGGGCGATGCCACC
TACGGCAAGCTGACCCTGAAGCTCATCTGCACCACCGGCAAGCTGCCCGTGCCCTGGCCCACCCTCGTGACC
ACCCTCGGCTACGGCCTGCAGTGCTTCGCCCGCTACCCCGACCACATGAAGCAGCACGACTTCTTCAAGTCC
GCCATGCCCGAAGGCTACGTCCAGGAGCGCACCATCTTCTTCAAGGACGACGGCAACTACAAGACCCGCGC
CGAGGTGAAGTTCGAGGGCGACACCCTGGTGAACCGCATCGAGCTGAAGGGCATCGACTTCAAGGAGGAC
GGCAACATCCTGGGGCACAAGCTGGAGTACAACTACAACAGCCACAACGTCTATATCACCGCCGACAAGCA
GAAGAACGGCATCAAGGCCAACTTCAAGATCCGCCACAACATCGAGGACGGCGGCGTGCAGCTCGCCGAC
CACTACCAGCAGAACACCCCCATCGGCGACGGCCCCGTGCTGCTGCCCGACAACCACTACCTGAGCTACCA
GTCCAAGCTGAGCAAAGACCCCAACGAGAAGCGCGATCACATGGTCCTGCTGGAGTTCGTGACCGCCGCCG
GGATCACTCTCGGCATGGACGAGCTGTACAAGGTATTTGAAGCCTCAGGAACAACTACTCAGGCCTACAGA
GTCGATGAAAGAGCCGCCGAGCAGGCTCGTTGGGAAGCAGCCTCCAAAGAAACCATCAAGAAAACCACCA
AGCCCTGTCCCCGCTGCCATGTACCAGTGGAAAAAAATGGAGGCTGCATGCACATGAAGTGTCCGCAGCCC
CAGTGCAGGCTCGAGTGGTGCTGGAACTGTGGCTGCGAGTGGAACCGCGTCTGCATGGGGGACCACTGGTT
CGACGTGTAG

Supplementary Figure 14 (continued)

FRET466

ATGGTGAGCAAGGGCGAGGAGCTGTTCACCGGGGTGGTGCCCATCCTGGTCGAGCTGGACGGCGACGTAAA
CGGCCACAAGTTCAGCGTGTCCGGCGAGGGCGAGGGCGATGCCACCTACGGCAAGCTGACCCTGAAGTTCA
TCTGCACCACCGGCAAGCTGCCCGTGCCCTGGCCCACCCTCGTGACCACCCTGACCTGGGGCGTGCAGTGCT
TCGCCCGCTACCCCGACCACATGAAGCAGCACGACTTCTTCAAGTCCGCCATGCCCGAAGGCTACGTCCAG
GAGCGCACCATCTTCTTCAAGGACGACGGCAACTACAAGACCCGCGCCGAGGTGAAGTTCGAGGGCGACAC
CCTGGTGAACCGCATCGAGCTGAAGGGCATCGACTTCAAGGAGGACGGCAACATCCTGGGGCACAAGCTG
GAGTACAACGCCATCAGCGACAACGTCTATATCACCGCCGACAAGCAGAAGAACGGCATCAAGGCCAACTT
CAAGATCCGCCACAACATCGAGGACGGCAGCGTGCAGCTCGCCGACCACTACCAGCAGAACACCCCCATCG
GCGACGGCCCCGTGCTGCTGCCCGACAACCACTACCTGAGCACCCAGTCCAAGCTGAGCAAAGACCCCAAC
GAGAAGCGCGATCACATGGTCCTGCTGGAGTTCGTGACCGCCGCCGGGATCACTCTCGGCATGGACGAGCT
GTACAAGTCCGGACTCAGATCTGGAGCAATGATAGTGTTTGTCAGGTTCAACTCCAGCCATGGTTTCCCAGT
GGAGGTCGATTCTGACACCAGCATCTTCCAGCTCAAGGAGGTGGTTGCTAAGCGACAGGGGGTTCCGGCTG
ACCAGTTGCGTGTGATTTTCGCAGGGAAGGAGCTGAGGAATGACTGGACTGTGCAGAATTGTGACCTGGAT
CAGCAGAGCATTGTTCACATTGTGCAGAGACCGTGGAGAAAAGGTCAAGAAATGAATGCAACTGGAGGCG
ACGACCCCAGAAACGCGGCGGGAGGCTGTGAGCGGGAGCCCCAGAGCTTGACTCGGGTGGACCTCAGCAG
CTCAGTCCTCCCAGGAGACTCTGTGGGGCTGGCTGTCATTCTGCACACTGACAGCAGGAAGGACTCACCAC
CAGCTGGAAGTCCAGCAGGTAGATCAATCTACAACAGCTTTTATGTGTATTGCAAAGGCCCCTGTCAAAGA
GTGCAGCCGGGAAAACTCAGGGTACAGTGCAGCACCTGCAGGCAGGCAACGCTCACCTTGACCCAGGGTCC
ATCTTGCTGGGATGATGTTTTAATTCCAAACCGGATGAGTGGTGAATGCCAATCCCCACACTGCCCTGGGAC
TAGTGCAGAATTTTTCTTTAAATGTGGAGCACACCCCACCTCTGACAAGGAAACATCAGTAGCTTTGCACCT
GATCGCAACAAATAGTCGGAACATCACTTGCATTACGTGCACAGACGTCAGGAGCCCCGTCCTGGTTTTCCA
GTGCAACTCCCGCCACGTGATTTGCTTAGACTGTTTCCACTTATACTGTGTGACAAGACTCAATGATCGGCA
GTTTGTCCACGACCCTCAGCTTGGCTACTCCCTGCCTTGTGTGGCTGGCTGTCCCAACTCCTTGATTAAAGAG
CTCCATCACTTCAGGATTCTGGGAGAAGAGCAGTACAACCGGTACCAGCAGTATGGTGCAGAGGAGTGTGT
CCTGCAGATGGGGGGCGTGTTATGCCCCCGCCCTGGCTGTGGAGCGGGGCTGCTGCCGGAGCCTGACCAGA
GGAAAGTCACCTGCGAAGGGGGCAATGGCCTGGGCTGTGGGTTTGCCTTCTGCCGGGAATGTAAAGAAGCG
TACCATGAAGGGGAGTGCAGTGCCGTATTTGAAGCCTCAGGAACAACTACTCAGGCCTACAGAGTCGATGA
AAGAGCCGCCGAGCAGGCTCGTTGGGAAGCAGCCTCCAAAGAAACCATCAAGAAAACCACCAAGCCCTGT
CCCCGCTGCCATGTACCAGTGGAAAAAAATGGAGGCTGCATGCACATGAAGTGTCCGCAGCCCCAGTGCAG
GCTCGAGTGGTGCTGGAACTGTGGCTGCGAGTGGAACCGCGTCTGCATGGGGGACCACTGGTTCGACGTGT
AAGCTTCGAATTCTGCAGTCGACGGTACCGCGGGCCCGGGATCCACCGGTCGCCACCATGGTGAGCAAGGG
CGAGGAGCTGTTCACCGGGGTGGTGCCCATCCTGGTCGAGCTGGACGGCGACGTAAACGGCCACAAGTTCA
GCGTGTCCGGCGAGGGCGAGGGCGATGCCACCTACGGCAAGCTGACCCTGAAGCTCATCTGCACCACCGGC
AAGCTGCCCGTGCCCTGGCCCACCCTCGTGACCACCCTCGGCTACGGCCTGCAGTGCTTCGCCCGCTACCCC
GACCACATGAAGCAGCACGACTTCTTCAAGTCCGCCATGCCCGAAGGCTACGTCCAGGAGCGCACCATCTT
CTTCAAGGACGACGGCAACTACAAGACCCGCGCCGAGGTGAAGTTCGAGGGCGACACCCTGGTGAACCGC
ATCGAGCTGAAGGGCATCGACTTCAAGGAGGACGGCAACATCCTGGGGCACAAGCTGGAGTACAACTACA
ACAGCCACAACGTCTATATCACCGCCGACAAGCAGAAGAACGGCATCAAGGCCAACTTCAAGATCCGCCAC
AACATCGAGGACGGCGGCGTGCAGCTCGCCGACCACTACCAGCAGAACACCCCCATCGGCGACGGCCCCGT
GCTGCTGCCCGACAACCACTACCTGAGCTACCAGTCCAAGCTGAGCAAAGACCCCAACGAGAAGCGCGATC
ACATGGTCCTGCTGGAGTTCGTGACCGCCGCCGGGATCACTCTCGGCATGGACGAGCTGTACAAGTAA

Supplementary Figure 14. Sequences of FRET reporter constructs. The complete sequences for each FRET reporter construct. Sequence corresponding to Cerulean is highlighted in aqua, Parkin highlighted in gray, Venus highlighted in Yellow, and linker regions not highlighted.

Supplementary Figure 15

Uncropped Images Related to Fig. 1

Fig. 1d: Mfn2

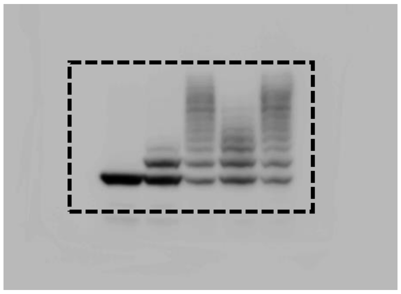

Fig. 1d: PDH

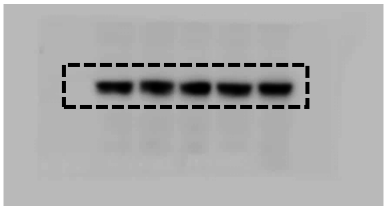

Fig. 1d: Ub

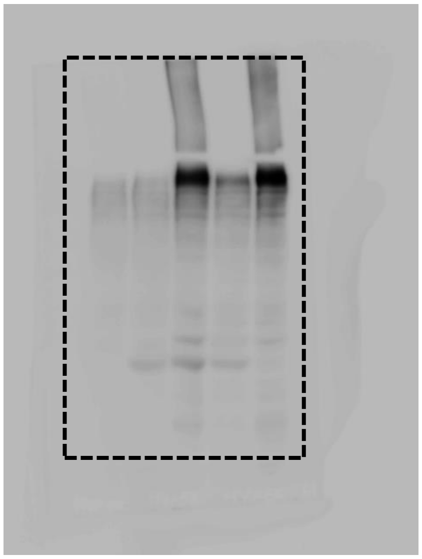

Fig. 1d: Parkin (CBB)

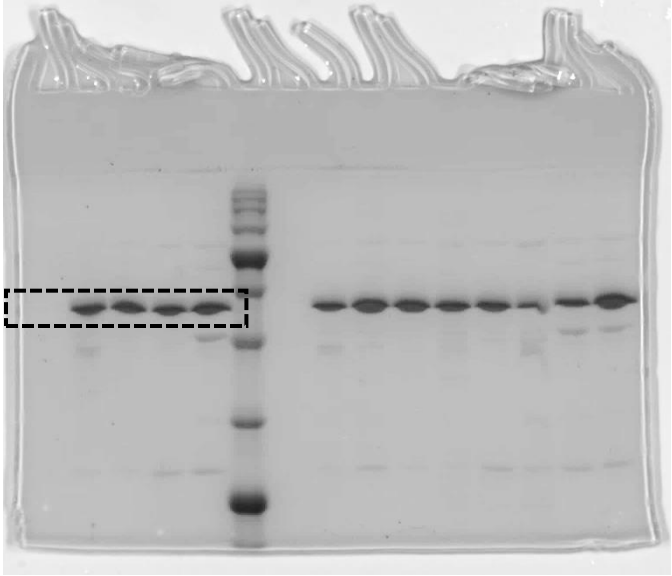

Supplementary Figure 15 (continued)

Uncropped Images Related to Fig. 2

Fig. 2c: Mfn2

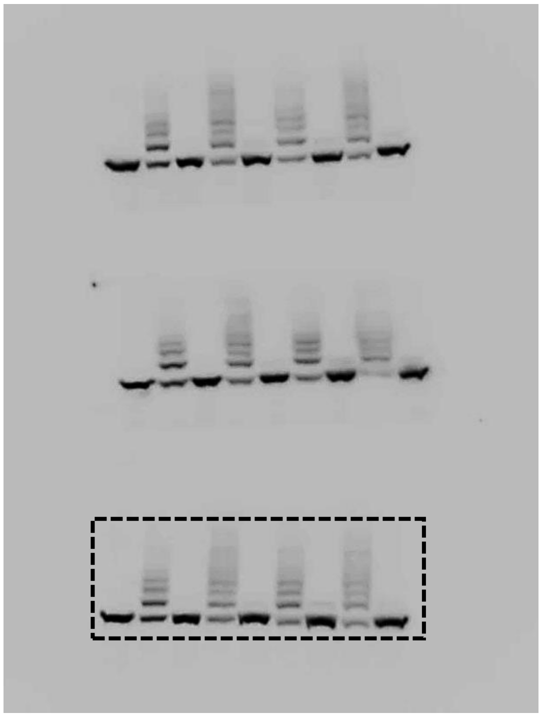

Fig. 2c: PDH

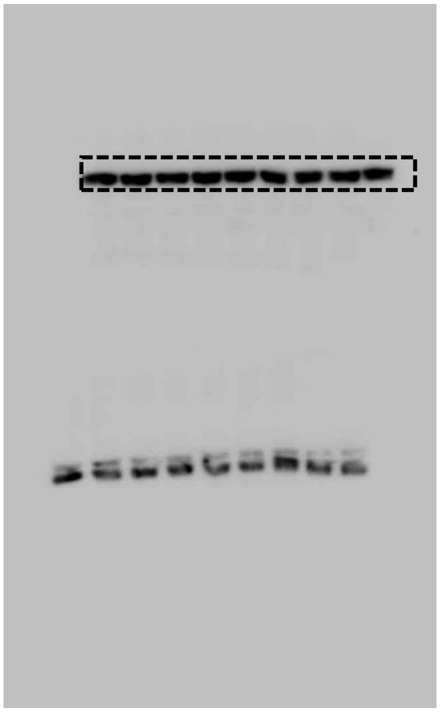

Fig. 2c: Parkin (CBB)

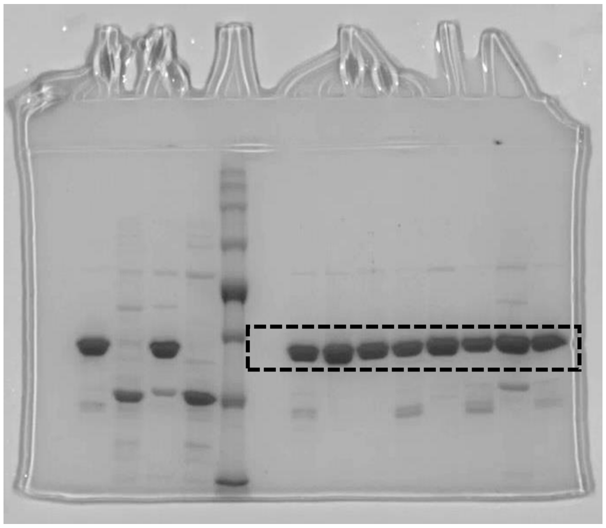

Supplementary Figure 15 (continued)

Uncropped Images Related to Fig. 2

Fig. 2e: Mfn2

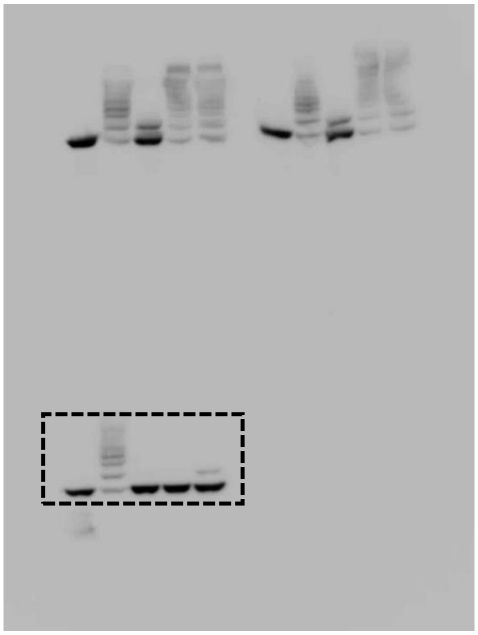

Fig. 2e: Parkin

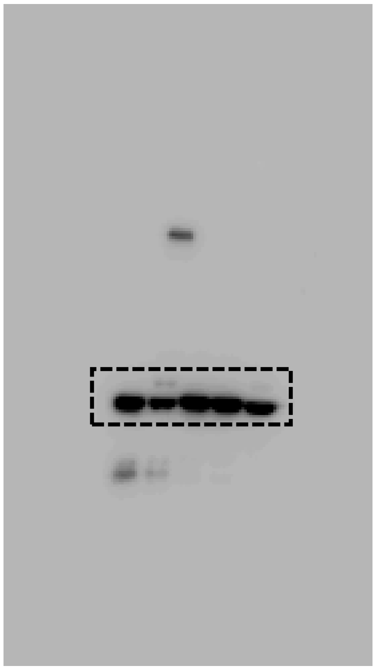

Fig. 2e: Parkin (Phostag)

Fig. 2e: Parkin (Phostag, CIP)

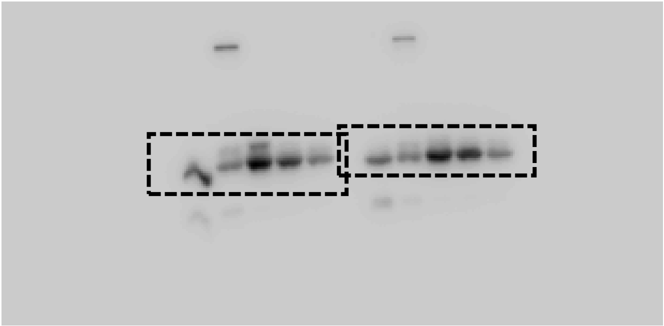

Fig. 2e: PDH

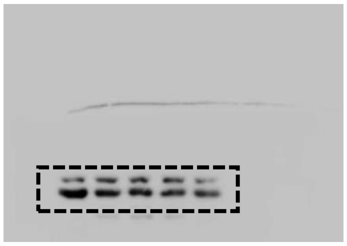

Supplementary Figure 15 (continued)

Uncropped Images Related to Fig. 3

Fig. 3c: Mfn2

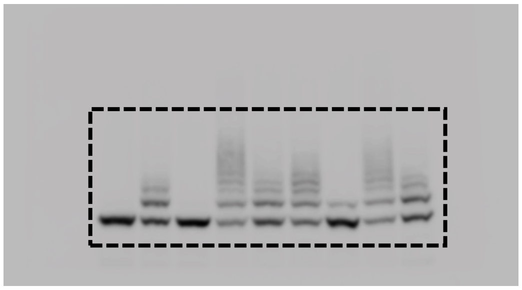

Fig. 3c: Parkin (CBB)

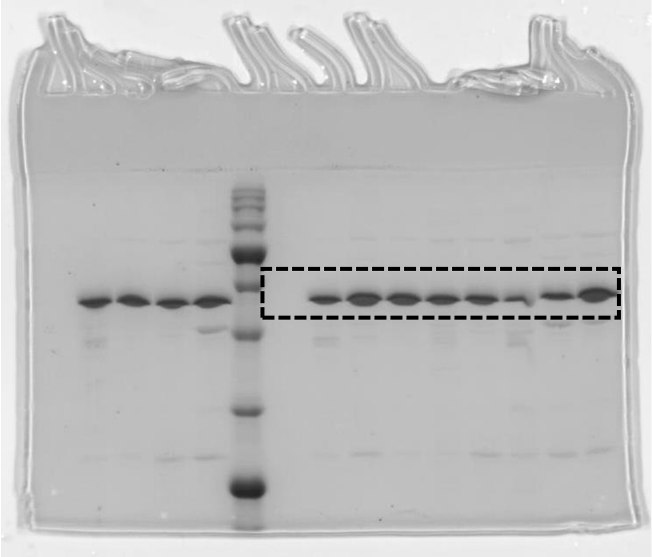

Fig. 3c: PDH

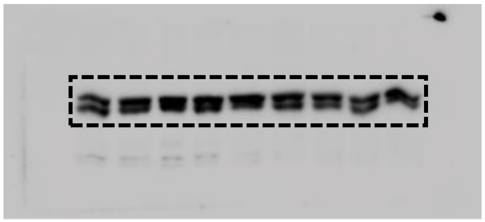

Supplementary Figure 15 (continued)

Uncropped Images Related to Fig. 4

Fig. 4c: Mfn2

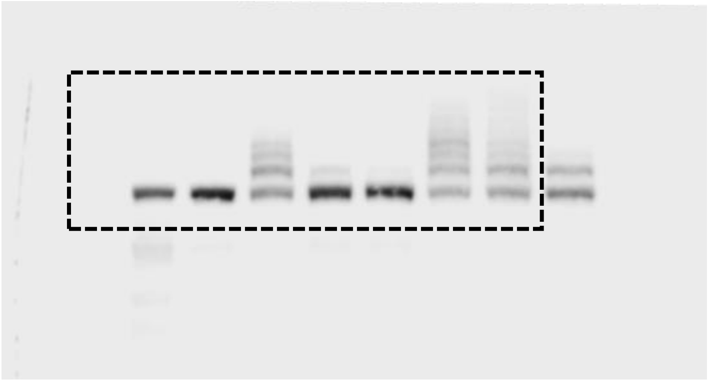

Fig. 4c: Parkin (CBB)

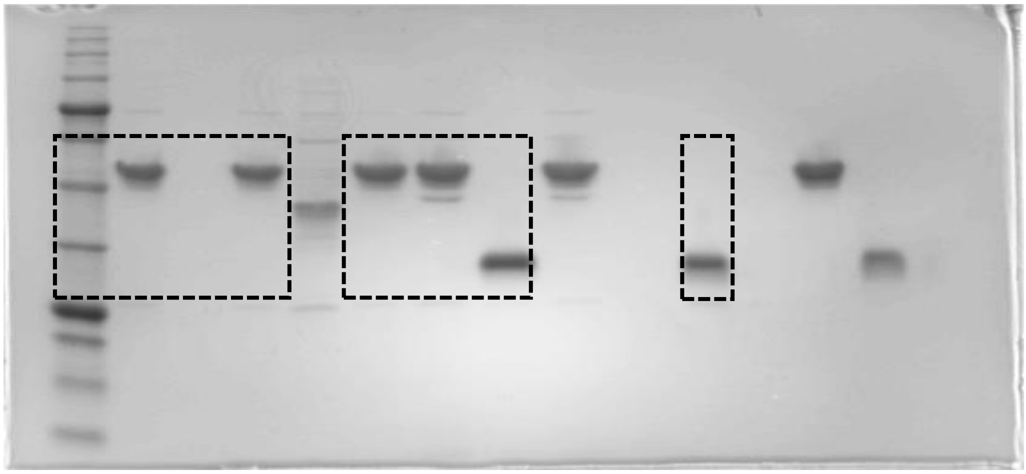

Fig. 4c: PDH

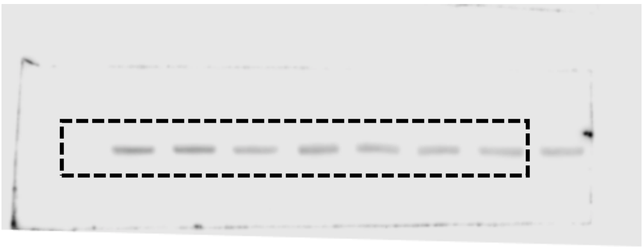

Supplementary Figure 15. Uncropped images corresponding to Fig 1d, Fig 2c, Fig 2e, Fig 3c, and Fig 4c.
